# Polyunsaturated fatty acid sequestration protects against mitochondrial dysfunction-induced ferroptosis

**DOI:** 10.1038/s44319-026-00898-y

**Published:** 2026-08-14

**Authors:** Guillermo Puertas-Frías, María José Saucedo-Rodríguez, Kristýna Čunátová, Lukáš Alán, Patrik Hrbáč, Michal Knězů, Soňa Koníčková, Jakub Schimmer, Monika Krakovková, Marek Vrbacký, Tomáš Čajka, Ondřej Kuda, Erika Fernández-Vizarra, Massimo Zeviani, Hana Hansíková, Tomáš Honzík, Josef Houštěk, Petr Pecina, Tomáš Mráček, Alena Pecinová

**Affiliations:** 1 Laboratory of Bioenergetics, Institute of Physiology, Czech Academy of Sciences, Vídeňská 1083, Prague, Czech Republic; 2 Faculty of Science, Charles University, 12800 Prague, Czech Republic; 3 Laboratory of Metabolism of Bioactive Lipids, Institute of Physiology, Czech Academy of Sciences, Vídeňská 1083, Prague, Czech Republic; 4 Laboratory of Metabolomics, Institute of Physiology, Czech Academy of Sciences, Vídeňská 1083, Prague, Czech Republic; 5 Department of Biochemistry and Molecular and Cellular Biology. Faculty of Health and Sport Sciences. University of Zaragoza, Zaragoza, Spain; 6 IRCCS Women and Children’s Hospital “Burlo-Garofolo”, Trieste, Italy; 7 Department of Paediatrics and Inherited Metabolic Disorders, First Faculty of Medicine, Charles University, General University Hospital, Prague, Czech Republic

**Keywords:** Autophagy & Cell Death, Metabolism, Molecular Biology of Disease

## Abstract

Impaired energy production is a hallmark of mitochondrial oxidative phosphorylation (OXPHOS) defects. However, secondary metabolic disturbances also represent an important trigger for pathologies originating from OXPHOS aberrations. Here we show that cells with OXPHOS deficiencies accumulate triacylglycerols enriched in polyunsaturated fatty acids (PUFAs), which are stored in lipid droplets. Sequestration of PUFAs is a critical component of a broader stress response, which also includes downregulation of cellular desaturases and upregulation of glutathione peroxidase 4 (GPX4). We demonstrate that this mechanism represents a physiologically relevant protective strategy, manifesting in cells under hypoxia and in immortalised fibroblasts derived from patients with primary mitochondrial complex IV deficiency. As a proof of principle, we observe elevated PUFA-enriched triacylglycerols in the plasma of patients with Myoclonic Epilepsy with Ragged Red Fibres (MERRF). Our findings reveal a novel protective mechanism against ferroptosis, which preserves membrane integrity when mitochondrial respiration is compromised.

## Synopsis

OXPHOS-deficient cells activate a coordinated multilayered polyunsaturated fatty acid (PUFA) stress response, which preserves membrane integrity against lipid peroxidation-driven ferroptosis. It is conserved across distinct OXPHOS complex deficiencies, is recapitulated under hypoxia, and is detectable in patients with mitochondrial disease.

- OXPHOS-deficient cells trigger a PUFA stress response that includes sequestration of PUFAs to triacylglycerols (TGs), downregulation of fatty acid desaturases, and upregulation of glutathione peroxidase 4.
- PUFA trafficking to TGs stored in lipid droplets protects the cells against lipid peroxidation and ferroptosis.
- OXPHOS-deficient cells synthesise fatty acids de novo from glutamine-derived acetyl-CoA when reductive carboxylation is permissible.
- PUFA stress response is activated in patients with mitochondrial deficiencies and during hypoxia.

## Abstract (web summary)

OXPHOS-deficient cells activate a coordinated multilayered polyunsaturated fatty acid (PUFA) stress response, which preserves membrane integrity against lipid peroxidation-driven ferroptosis. It is conserved across distinct OXPHOS complex deficiencies, is recapitulated under hypoxia, and is detectable in patients with mitochondrial disease.

## Graphical abstract

**Figure:**
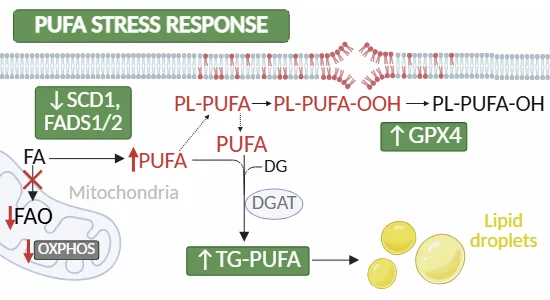

## Introduction

Mitochondrial oxidative phosphorylation (OXPHOS), a key metabolic pathway, generates cellular ATP by mitochondrial ATP synthase, which utilises the electrochemical gradient formed by the electron transport system (ETS). OXPHOS also provides biosynthetic intermediates and maintains NAD^+^/NADH balance (Yusri et al, 2025). In humans, OXPHOS defects constitute a group of severe inborn errors of metabolism (Fernandez-Vizarra and Zeviani, 2021). Their biochemical consequences depend on the affected OXPHOS component, but commonly include decreased ATP production, accumulation of NADH, and potentially elevated reactive oxygen species (reviewed in (Martinez-Reyes and Chandel, 2020; Smeitink et al, 2006)). Accumulated NADH directly affects the tricarboxylic acid (TCA) cycle, glycolysis, malate-aspartate shuttle, and de novo aspartate synthesis, which is essential for proliferation (Birsoy et al, 2015; Mullen et al, 2011; Sullivan et al, 2015).

To survive, cells with compromised OXPHOS need to reprogram their metabolism. Reliance on substrate-level phosphorylation during glycolysis or glutamine oxidation can partially compensate for impaired ATP production (Cunatova et al, 2024; Chen et al, 2018; Kiss et al, 2014; Sturm et al, 2023), while NAD^+^ regeneration requires an alternative electron acceptor, such as pyruvate, which is converted to lactate (King and Attardi, 1989; Sullivan et al, 2015). Several groups further demonstrated that uridine is required for pyrimidine nucleotide synthesis to support proliferation when electrons cannot be transported through ETS (Adant et al, 2022; King and Attardi, 1989) to bypass dihydroorotate dehydrogenase (Adant et al, 2022; Sullivan et al, 2015). Cells with impaired ETS may also redirect glutamine-derived α-ketoglutarate to citrate via reductive carboxylation. This process is regulated by altering the α-ketoglutarate/citrate ratio (Fendt et al, 2013), but a low NAD(P)^+^/NAD(P)H ratio may also facilitate the process (Mullen et al, 2011). It was demonstrated that reductive carboxylation is coupled to glycolysis via cytosolic malate dehydrogenase 1 (Gaude et al, 2018). Additionally, desaturase-mediated NAD^+^ recycling has been proposed as an acute adaptation to impaired respiration (Kim et al, 2019).

While adaptive control of redox balance and ATP supply is well documented, the downstream consequences are less understood. Cells with compromised OXPHOS, due to hypoxia or mitochondrial DNA depletion, form lipid droplets (LDs) (Lee et al, 2013). LDs are the lipid storage organelles that respond to stress stimuli, such as nutrient and oxidative stress, by membrane remodelling (Danielli et al, 2023). Membrane phospholipids containing polyunsaturated fatty acids (PUFAs) are more prone to lipid peroxidation, which may ultimately lead to ferroptosis, a type of iron-dependent regulated cell death (Dixon et al, 2012). Sequestration of PUFAs from membrane phospholipids into neutral triacylglycerols (TGs) stored in LDs has been shown in tumours (Dierge et al, 2021; Jarc et al, 2018), in *D. melanogaster* stem cells exposed to hypoxia (Bailey et al, 2015) or during cell-cycle arrest (Lee et al, 2024). Conversely, lipid starvation drives PUFAs trafficking from TGs to phospholipids and promotes ferroptosis in tumour cells (Sokol et al, 2025).

Because OXPHOS function is tightly coupled to fatty acid oxidation, we studied lipid metabolism remodelling as a survival strategy when OXPHOS is limited, using cellular models deficient in complex II (CII), complex IV (CIV), or complex V (CV). To assess the universality of our findings, we also studied control cells exposed to hypoxia and patients with mitochondrial disease. We show that cells with compromised OXPHOS activate a PUFA stress response that involves sequestration of PUFAs into neutral lipids, reduced expression of cellular desaturases, and upregulation of the antioxidant phospholipid peroxidase GPX4.

## Results and discussion

### CIV-deficient cells accumulate succinate derived from glutamine

To investigate metabolic alterations that allow cell survival without respiration, we used three HEK293-derived cell lines – wild-type (WT), COX6B1 knock-out (6BKO), and 6BKO cells overexpressing alternative oxidase (6BAOX) (Cunatova et al, 2024). AOX accepts electrons directly from the coenzyme Q pool, partially restoring ETS flux upstream of CoQ (Appendix Fig. 1A). 6BKO cells lack assembled cytochrome c oxidase (CIV) and show secondary complex I reduction, abolished respiration and decreased NAD^+^/NADH ratio, all partially rescued by AOX (Cunatova et al, 2026; Cunatova et al, 2024). Mitochondrial ROS levels remained unaltered, and ATP demands were sustained by upregulated glycolysis (Cunatova et al, 2024).

**Figure 1:**
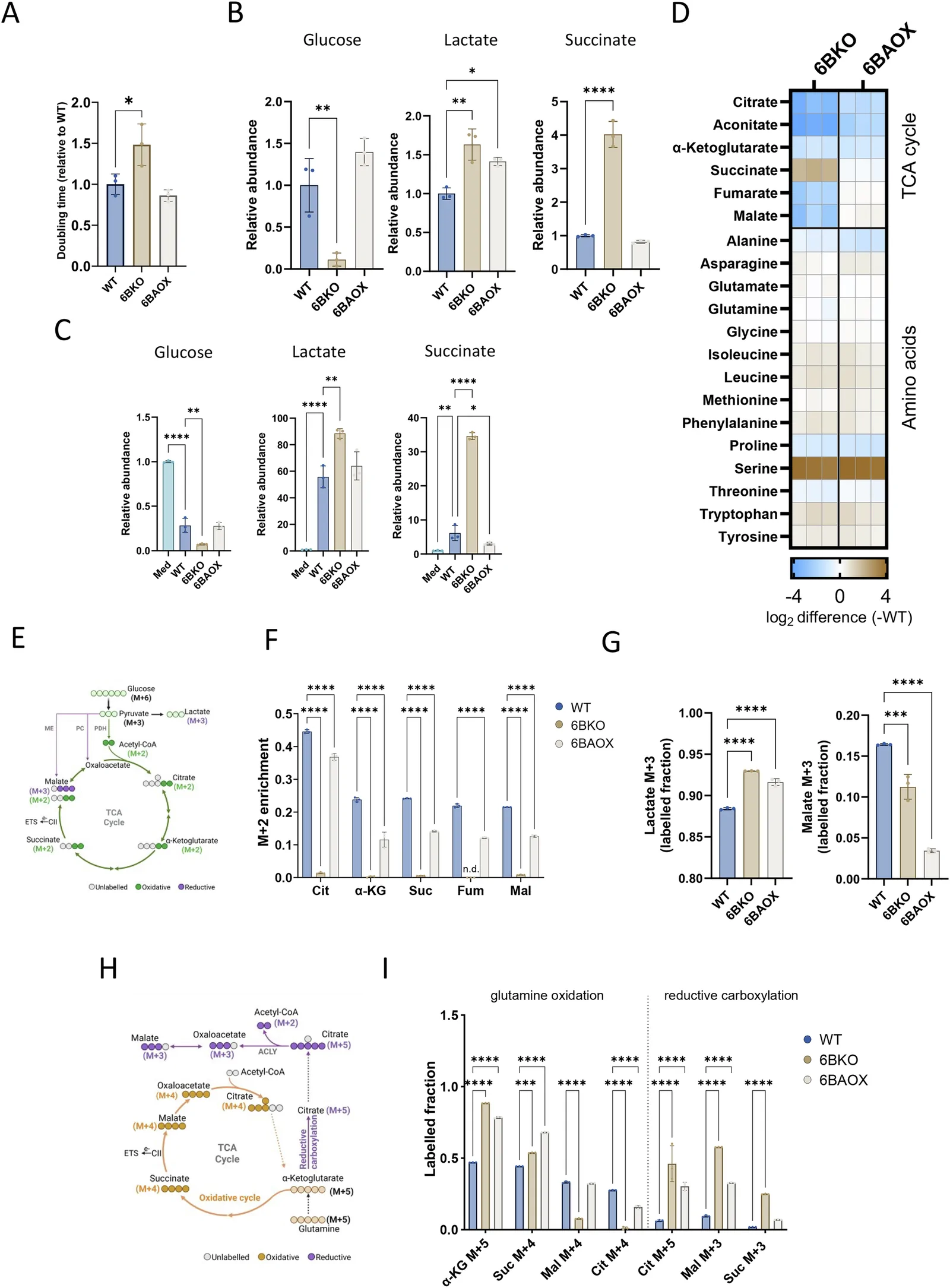
CIV deficiency promotes remodelling of the mitochondrial metabolism. (**A**) Doubling time (relative to WT) of the cell lines measured by the CyQuant proliferation kit (*n* = 3). Metabolic profiling of glucose, lactate and succinate in the cells (**B**) and medium (**C**) after 24 h incubation with fresh culture media. Data are expressed as fold changes to WT (**B**) or fresh media (**C**). (**D**) Heatmap showing differences in TCA cycle metabolites and amino acid levels in 6BKO and 6BAOX cells after 24 h of incubation in culture media. Data represent log_2_(fold change) compared to WT (*n* = 3). Schematic illustration of the metabolite labelling pattern from ^13^C_6_-glucose (**E**) or ^13^C_5_-glutamine (**H**). M+ indicates the number of ^13^C atoms (coloured) after 24 h incubation with the tracer. Labelled fraction (proportion of total pool) of M + 2 TCA cycle metabolites (**F**) and M + 3 lactate and malate (**G**) derived from ^13^C_6_-glucose. (**I**) Labelled fraction of the TCA cycle metabolites originating from oxidation or reductive carboxylation of ^13^C_5_-glutamine. Cit citrate, α-KG α-ketoglutarate, Suc succinate, Fum fumarate, Mal malate, n.d. not detected. Data were expressed as fold changes compared to WT (*n* = 3). *N* represents the number of biological replicates. Bar graphs represent means ± SD. One-way (**A**–**C**, **G**) or two-way (**F**, **I**) ANOVA was performed (**A** - p* = 0.0127; **B** - p** = 0.0038 (glucose), 0.016 (lactate), p* = 0.0128; **C** – p* = 0.0353, p** = 0.0012 (glucose), 0.0023(succinate); **B**, **C**, **F**, **G**, **I** - p**** <0.0001). Source data are available online for this figure.

**Figure EV1:**
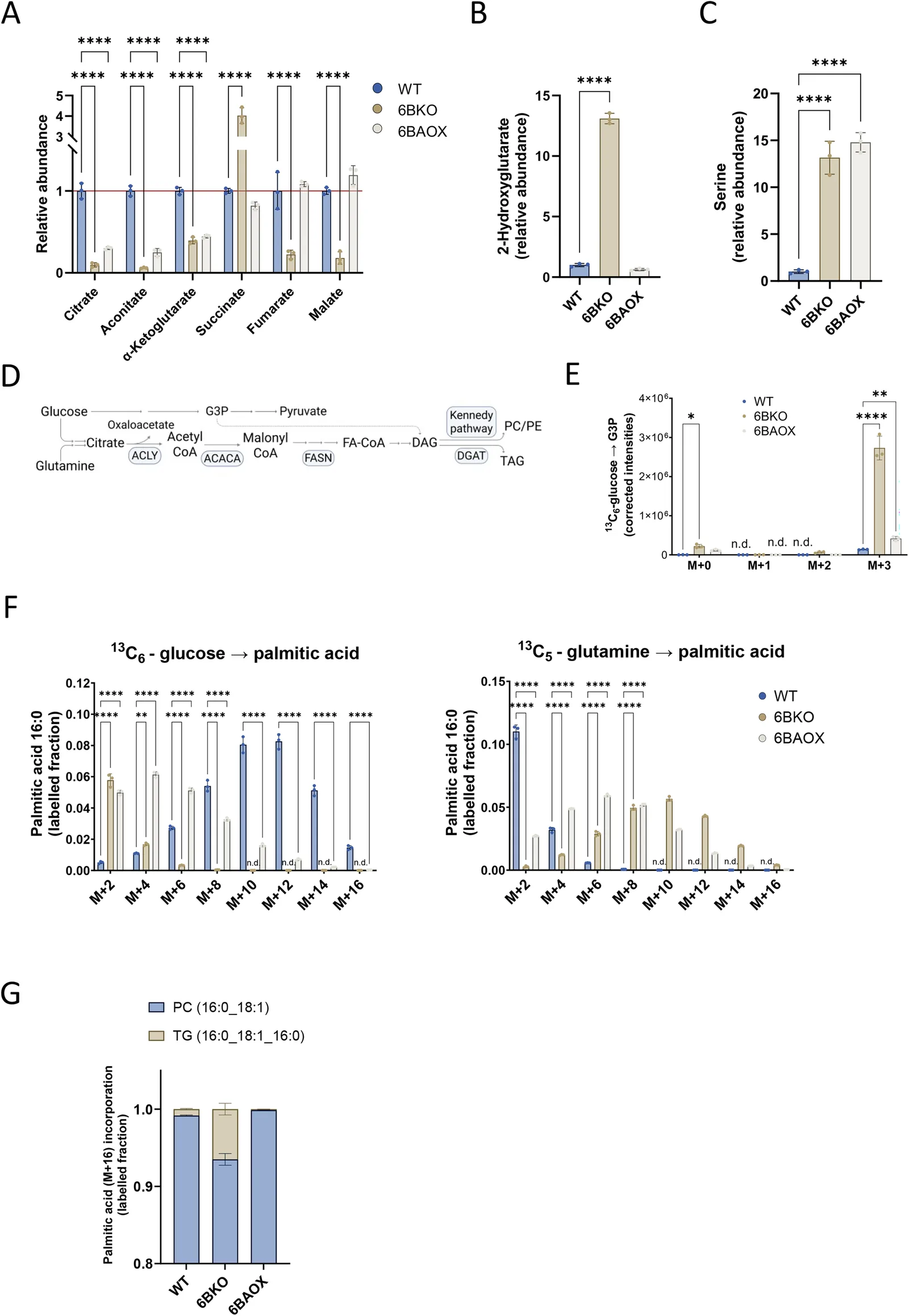
Metabolic remodelling in CIV-deficient cells. Metabolic profiling (LC–MS) of TCA cycle metabolites (**A**), 2-hydroxyglutarate (**B**), and serine (**C**) after 24 h incubation in fresh culture media. Data are expressed as fold changes compared to WT (*n* = 3). (**D**) Schematic representation of de novo lipid synthesis. (**E**) Glycerol 3-phosphate (G3P) labelling from ^13^C_6_-glucose after 24 h incubation (*n* = 3). (**F**) ^13^C labelling of palmitic acid after 24 h incubation with ^13^C_6_-glucose or ^13^C_5_-glutamine measured by LC–MS after fatty acyl chain lipolysis (*n* = 3). (**G**) Metabolic modelling of the flux of de novo synthesised palmitic acid into TG (1-palmitoyl-2-oleoyl-sn-glycero-3-phosphocholine; TG 16:0_18:1_18:1) or phosphatidylcholine (PC 16:0_18:1). Data were expressed as fold changes relative to control (*n* = 6). *N* represents the number of biological replicates. Bar graphs represent means ± SD. One-way (**B**, **C**) or two-way (**A**, **F**) ANOVA was performed (**A**–**C** – p**** < 0.0001; **E** – p* = 0.0124, p** = 0.0017; **F** – p* = 0.0024; p**** < 0.0001).

Doubling time was significantly longer in 6BKO cells than in WT and 6BAOX (Fig. 1A). LC–MS analysis revealed higher glucose utilisation and lactate accumulation in 6BKO, both intracellularly (Fig. 1B) and in the medium (Fig. 1C), confirming increased ATP production by glycolytic substrate-level phosphorylation (Cunatova et al, 2024). Succinate was strongly accumulated in 6BKO cells and media, yet normalised by AOX (Figs. 1B,C and EV1A). Upstream TCA metabolites (citrate, aconitate and α-ketoglutarate) were significantly decreased in both 6BKO and 6BAOX, while downstream ones (fumarate, malate) were lower in 6BKO and restored by AOX (Figs. 1D and EV1A). 2-hydroxyglutarate, recently shown to rise in CLPP-deficient cells (Kaul et al, 2025), was elevated in 6BKO cells (Fig. EV1B). Amino acids were comparable to WT, except for accumulated serine (Figs. 1D and EV1C) and aspartate, essential for nucleotide synthesis (Birsoy et al, 2015; Sullivan et al, 2015), which was very low, often below the detection limit.

We hypothesised that impaired OXPHOS triggers TCA cycle rewiring towards glutaminolysis, thereby replenishing TCA metabolite pools. To test this, we performed fluxomic analysis with ^13^C_6_-glucose or ^13^C_5_-glutamine (Fig. 1E,H). In glucose tracing, the M + 2-enriched fraction of all TCA metabolites was reduced in 6BKO, indicating a blocked canonical TCA cycle, which was partially restored by AOX (Fig. 1F). A higher M + 3 lactate fraction in 6BKO and 6BAOX (Fig. 1G) reflects faster pyruvate-to-lactate conversion and NADH reoxidation. Reduced M + 3 malate fraction in 6BKO and 6BAOX indicates a lesser metabolic flux via pyruvate carboxylase (PC) or malic enzyme (ME) 1/2 (Fig. 1G). The tracing analysis of glutamine revealed that the M + 5 α-KG fraction comprised almost the entire pool in 6BKO and 6BAOX, and succinate M + 4 was increased (Fig. 1I), showing oxidative glutamine metabolism that permits substrate-level phosphorylation by succinyl-CoA ligase (Seyfried et al, 2020). In 6BKO cells, malate M + 4 and citrate M + 4 were reduced, indicating that the TCA cycle stalls at the succinate level, and AOX expression restored succinate oxidation. A highly enriched citrate M + 5 fraction in 6BKO and lower 6BAOX, together with elevated M + 3 malate and succinate, indicates reductive carboxylation of α-KG to citrate, followed by its cytosolic conversion to malate (via ATP-citrate synthase (ACLY) and malate dehydrogenase (MDH)). Malate can re-enter the TCA cycle in reverse (e.g., in exchange for citrate (Palmieri et al, 2015)) to form succinate M + 3 (Fig. 1I).

Thus, CIV-null cells use glutamine as the alternative carbon source for the TCA cycle, accumulating succinate and 2-hydroxyglutarate, while glucose is preferentially converted to lactate. These bioenergetic hallmarks (impaired aerobic glycolysis, succinate accumulation, serine elevation and reductive glutamine carboxylation) align with prior studies of CII, CIII and CV deficiency (Cardaci et al, 2015; Gaude et al, 2018; Chen et al, 2018; Lussey-Lepoutre et al, 2015; Mullen et al, 2011). The serine accumulation observed across all our models likely reflects impaired NAD^+^-dependent catabolism (Yang et al, 2020) and possibly its protective de novo synthesis (Jackson et al, 2025).

### CIV-deficient cells accumulate triacylglycerols enriched for polyunsaturated fatty acids

**Figure 2:**
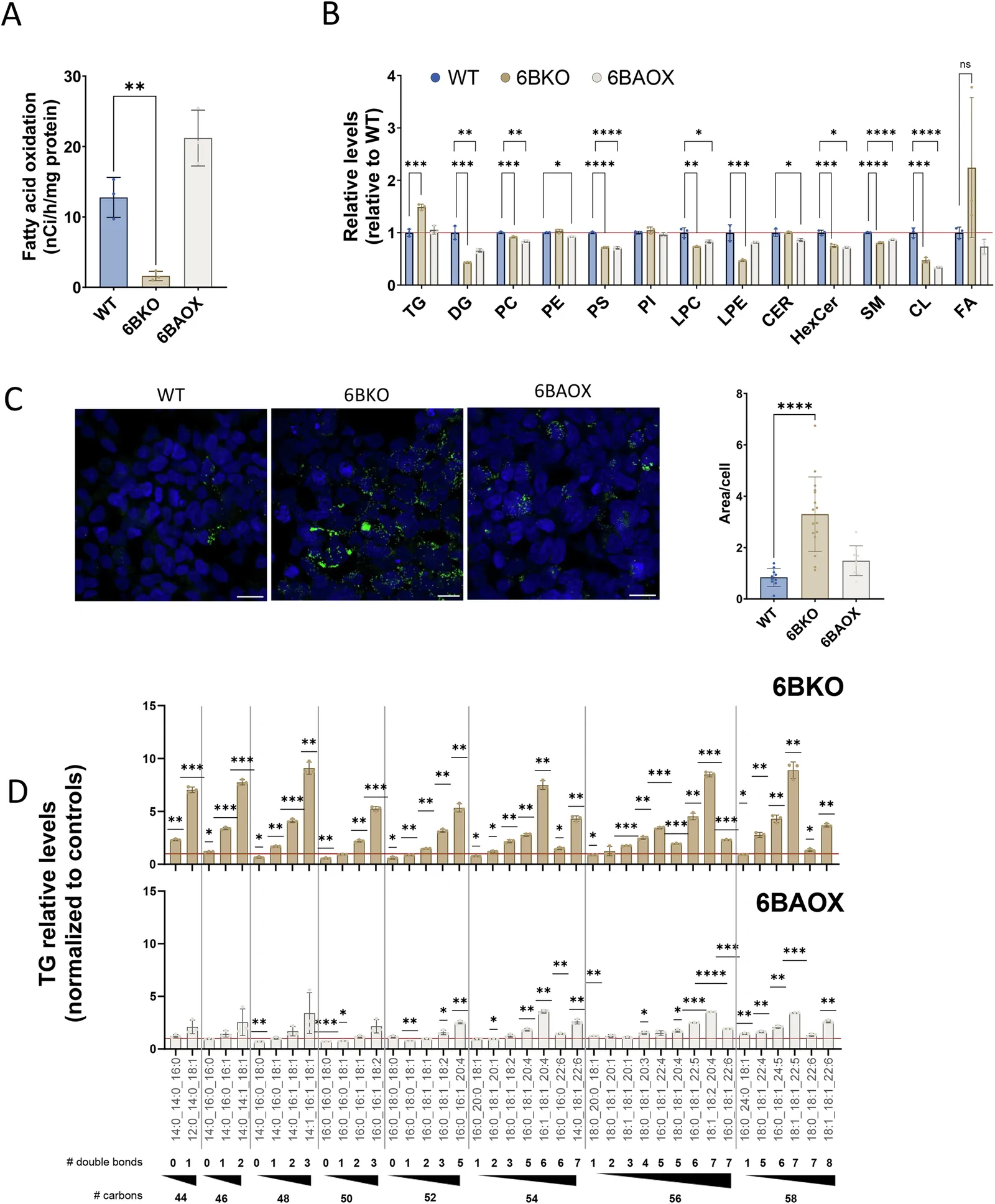
CIV-deficient cells accumulate TGs enriched for PUFAs. (**A**) Fatty acid oxidation of [9,10(n)-^3^H] palmitic acid (*n* = 3). (**B**) Relative levels of lipid classes measured by LC–MS (*n* = 3). TG triacylglycerols, DG diacylglycerols, PC phosphatidylcholines, PE phosphatidylethanolamines, PS phosphatidylserines, PI phosphatidylinositols, LPC lysophosphatidylcholines, LPE lysophosphatidylethanolamines, Cer ceramides, HexCer hexosylceramides, SM sphingomyelins, CL cardiolipins, FA fatty acids. Data were expressed as fold change to WT (*n*  = 3). (**C**) Representative image and quantification of BODIPY 493/503 staining of the cell lines (*n* ≥ 10). Scale bar size is 20 µm. (**D**) Analysis of relative intracellular TGs measured by LC–MS differentiated based on the total acyl chains carbons and content of double bonds in 6BKO and 6BAOX. Data were expressed as mean ± SD (*n* = 3). *N* represents the number of biological replicates. Bar graphs represent means ± SD. One-way ANOVA (**A**, **C**), two-way ANOVA (**B**) or one-sample *t*-test (**D**) was performed (**A** – p** = 0.0053; **B** – p* = 0.013 (PE), 0.0195 (LPC), 0.0156 (CER); p** = 0.0026 (DG), 0.0031 (PC), 0.0024 (LPC); p*** = 0.0003 (TG), 0.0002 (DG), 0.0006 (LPE), 0.0002 (HexCer, WT vs 6BKO), 0.0001 (HexCer, WT vs 6BAOX); **D** – see Table EV1; p**** <0.0001). Source data are available online for this figure.

ACLY-mediated conversion of citrate to oxaloacetate yields acetyl-CoA for de novo lipid synthesis. Since respiration is impaired in 6BKO, this also impaired fatty acid oxidation (FAO), which was restored by AOX (Fig. 2A). Together, this should lead to fatty acid accumulation. Lipidomic analysis showed significantly increased triacylglycerols (TGs) and decreased diacylglycerols in 6BKO compared to WT (Fig. 2B), with phospholipids comparable or slightly decreased and a non-significant rise in free fatty acids (Fig. 2B). Bodipy staining confirmed TGs storage in lipid droplets (LDs) (Olzmann and Carvalho, 2019) (Fig. 2C). Glycerol 3-phosphate (G3P), essential for TG synthesis (Fig. EV1D) was mainly glucose-derived and maintained higher in 6BKO (Fig. EV1E). AOX normalised all dysregulated features of TG metabolism (Figs. 2B,C and EV1E).

To uncover the origin of acetyl-CoA used for fatty acid de novo synthesis, we performed fluxomic analysis of fatty acyl chains. It showed that in WT, acetyl-CoA for de novo palmitate (16:0) or stearate (18:0) synthesis was derived mainly from glucose, whereas in 6BKO it was derived mainly from glutamine, with 6BAOX using both sources (Fig. EV1F; Appendix Fig. S1B). This coupling of reductive glutamine metabolism to lipogenesis forms a metabolic loop that simultaneously disposes of cytosolic acetyl-CoA and feeds TG synthesis. Metabolic modelling of de novo palmitate (16:0 M + 16) flux showed near-exclusive incorporation into phospholipids in WT and 6BAOX, but approximately 6% of de novo palmitate was deposited into TG in 6BKO (Fig. EV1G).

Because fatty acid desaturation and their storage in TGs have been linked to glycolytic NAD^+^ recycling upon acute complex I inhibition (Kim et al, 2019) and 6BKO has a low NAD^+^/NADH ratio (Cunatova et al, 2024), we examined the distribution of polyunsaturated fatty acids (PUFAs) in TGs. TGs with a higher number of double bonds were strongly elevated in 6BKO, up to 9-fold for specific species (Fig. 2D), and partially mitigated by AOX. Thus, in CIV-deficient cells, fatty acids are not oxidised but are synthesised de novo from glutamine-derived carbons and stored in lipid droplets as PUFA-enriched TGs.

### CIV-deficient cells activate PUFA-mediated stress response

**Figure EV2:**
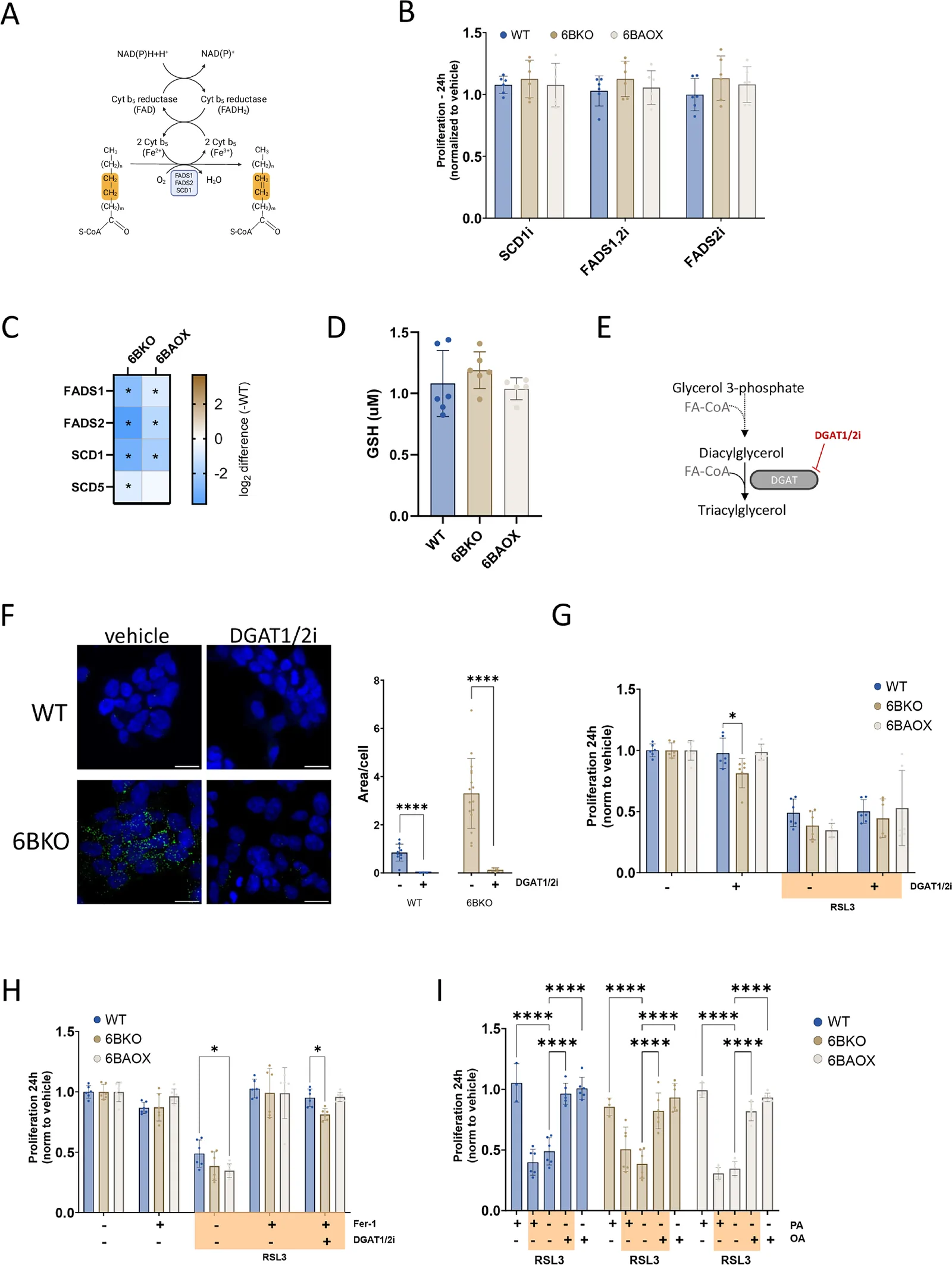
PUFA stress response in CIV-deficient cells. (**A**) Scheme of fatty acyl chain desaturation. (**B**) Cell proliferation analysis of the cell lines incubated for 24 h with inhibitors against SCD1 (CAY10566, 500 nM), FADS1 and 2 (CP-24879, 30 µM) and FADS2 (SC-26196, 10 µM). Data were expressed as fold change to vehicle (*n* = 6). (**C**) LFQ-MS analysis of the cellular desaturase levels. The heatmap displays log_2_(fold change); asterisks indicate proteins that are significantly altered (*p* value <0.05, *n* = 3). (**D**) GSH level assessed in WT, 6BKO and 6BAOX (*n* = 6). Data were expressed as fold change relative to WT. (**E**) Schematic representation of TG formation. (**F**) Representative image and quantification of BODIPY 493/503 staining in WT and 6BKO cell line treated with vehicle or a mixture of DGAT1 (A922500, 1 µM) and DGAT2 (PF-06424439, 10 µM) inhibitors (DGAT1/2i) (n ≥ 3). Scale bar size is 20 µm. (**G**–**I**) Proliferation analysis of the cell lines incubated for 24 h with RSL3 (1 µM). The cells were pretreated for 48 h with DGAT1/2i (**G**, **H**), or for 24 h with the ferroptosis inhibitor ferrostatin-1 (Fer-1, 200 nM) (**H**), BSA-conjugated oleic (OA, 25 µM) or palmitic (PA, 25 µM) acid (**I**). Data were expressed as fold change to vehicle (*n* = 6). *N* represents the number of biological replicates. Bar graphs represent means ± SD. Two-way (**B**, **G**–**I**), one-way (**D**) ANOVA, or *t*-test (**F**) was performed (**F**– p****0.0001; **G** – p* = 0.0350; **H** – p* = 0.0203 (RSL3), 0.0240 (RSL3 + DGAT1/2i+Fer-1); **I** – p**** < 0.0001).

We first tested whether desaturation of acyl chains recycles NAD^+^ to sustain glycolysis (Fig. EV2A). If so, inhibition of desaturases should harm 6BKO cells. Inhibitors of stearoyl-CoA desaturase 1 (SCD1, inhibitor CAY10566), acyl-CoA (8-3)-desaturase (FADS1, inhibitor CP-24879) and acyl-CoA 6-desaturase (FADS2, inhibitors CP-24879 and SC-26196) did not affect proliferation of 6BKO or 6BAOX at 24 h (Fig. EV2B) or 48 h (Appendix Fig. S1C). Label-free quantification mass spectrometry (LFQ-MS) analysis showed strongly decreased levels of the desaturases (up to 13-fold) in both cell lines (Fig. EV2C). This contrasts with the acute response to OXPHOS inhibition, in which desaturase activity transiently rises to support glycolytic NAD^+^ recycling (Kim et al, 2019). While the short-term PUFA formation may resolve reductive stress, sustained desaturation would create an unmanageable peroxidation burden. Our data indicate that OXPHOS-deficient cells resolve this conflict by progressively suppressing desaturases, while other enzymes, such as lactate dehydrogenase (King and Attardi, 1989; Sullivan et al, 2015), or malate dehydrogenase 1 (Gaude et al, 2018) maintain a survival-permissive redox balance. PUFA-rich TG accumulation should therefore not be considered as a NAD^+^-recycling mechanism in 6BKO.

**Figure 3:**
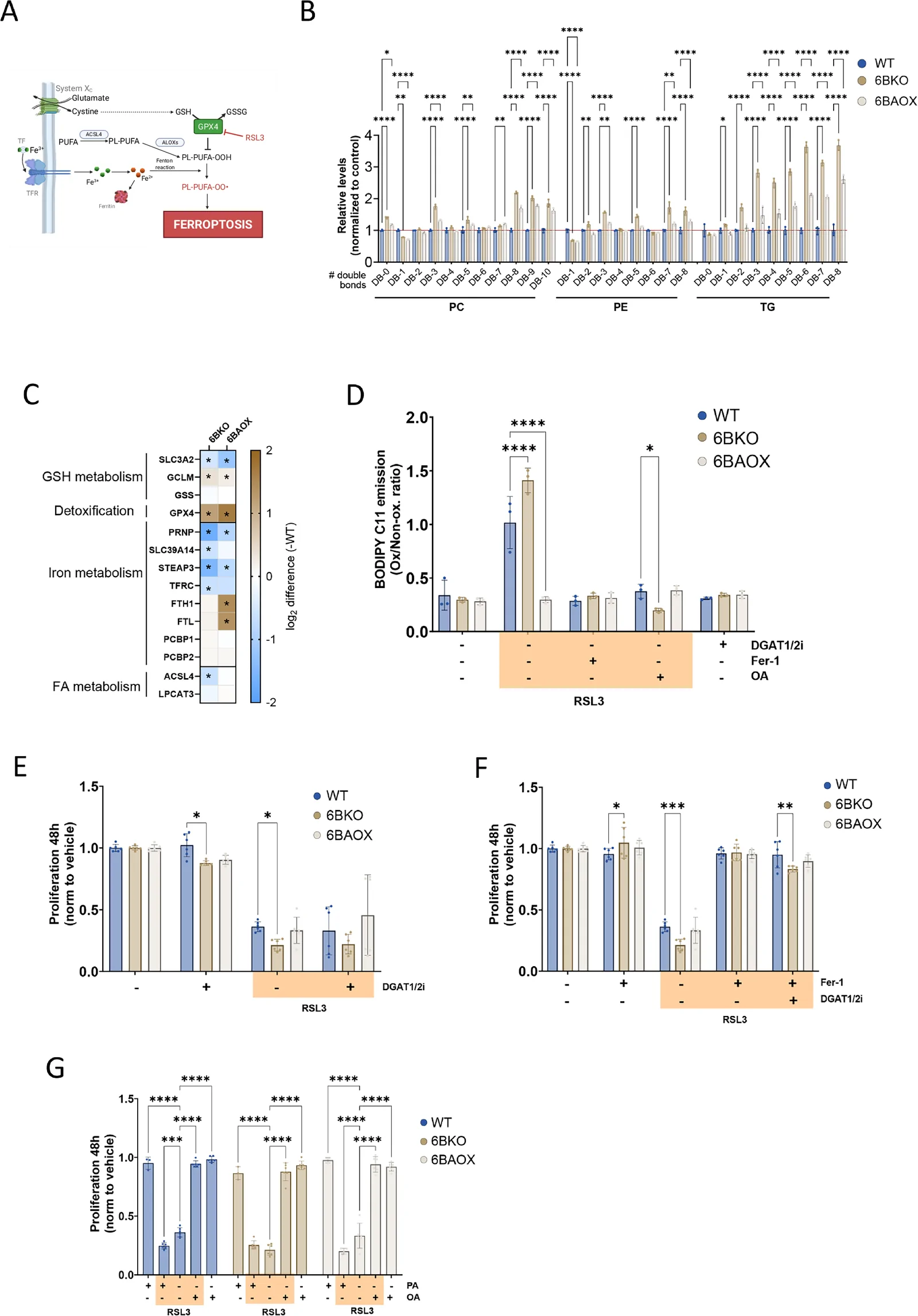
PUFA stress response is activated in CIV-deficient cells. (**A**) Schematic illustration of ferroptosis (according to the KEGG pathway hsa04216). (**B**) Relative levels of phospholipids (PC, PE) and TGs measured by LC–MS were differentiated based on double bond content. Data are expressed as fold change to WT (*n* = 3). (**C**) LFQ-MS analysis of ferroptosis-related protein levels. The heatmap displays log_2_(fold change); asterisks indicate proteins that are significantly altered (*n* = 3, *p* value <0.05). (**D**) Lipid peroxidation assessed by lipid peroxidation sensor BODIPY 581/591 C11 (*n* = 3) in the cell lines incubated for 24 h with a ferroptosis inducer RSL3 (1 µM). The cells were pretreated for 48 h with a mixture of DGAT1 (A922500, 1 µM) and DGAT2 (PF-06424439, 10 µM) inhibitors (DGAT1/2i), for 24 h with the ferroptosis inhibitor ferrostatin-1 (Fer-1, 200 nM), or with BSA-conjugated oleic acid (OA, 25 µM). (**E**–**G**) Proliferation analysis of the cell lines incubated for 48 h with RSL3 (1 µM). The cells were pretreated for 48 h with DGAT1/2i (**E**, **F**), or for 24 h with the ferroptosis inhibitor Fer-1 (**F**), or BSA-conjugated OA or PA (palmitic acid, 25 µM) (**G**). Data were expressed as fold change to vehicle (*n* ≥ 3). *N* represents the number of biological replicates. Bar graphs represent means ± SD. Two-way ANOVA was performed (**B** – see Table EV2; **D** - p* = 0.0273; **E** – p* = 0.0440 (DGAT1/2i), 0.0382 (RSL3); **F** – p* = 0.0141, p** = 0.0025, p*** = 0.0001; p**** < 0.0001). Source data are available online for this figure.

Because higher acyl chain desaturation increases the risk of membrane lipid peroxidation and ferroptosis (Fig. 3A) (Rodencal and Dixon, 2023; von Krusenstiern et al, 2023), we compared PUFA levels in phosphatidylcholines (PC) and phosphatidylethanolamines (PE) with TGs (Fig. 3B; Appendix Fig. S2A). Although some highly unsaturated phospholipids rose up to 40% in 6BKO, PUFA-enriched TGs increased up to fourfold. Free PUFAs, but not saturated or monounsaturated, were elevated in 6BKO (Appendix Fig. S2A), which is consistent with PUFA trafficking away from the membrane phospholipids. AOX attenuated both free PUFAs and their sequestration to TGs. PUFA-containing phospholipids are the primary substrates for peroxidation and ferroptosis (Reimers et al, 2023; Yang et al, 2016), even when total PUFA levels are low (Sokol et al, 2025), so this redistribution protects the membrane from becoming a substrate for iron-catalysed chain reactions.

The central enzyme eliminating PUFA-derived peroxyl radicals is glutathione peroxidase 4 (GPX4), which uses reduced glutathione (GSH) (Fig. 3A, (Rodencal and Dixon, 2023)). LFQ-MS analysis of the ferroptotic pathway (KEGG hsa04216) revealed significantly increased GPX4 and inconsistent changes in proteins involved in GSH synthesis in 6BKO and 6BAOX (Fig. 3C), while GSH level remained unchanged (Fig. EV2D). Iron import proteins (TFRC or SLC39A14) and long-chain-fatty acid--CoA ligase 4 (ACSL4), which activates PUFAs for membrane re-acylation by lysophospholipid acyltransferase 5 (LPCAT3), were decreased (Fig. 3C). This supports the view that the excess of PUFA-enriched membrane phospholipids represents a risk for the cell. We term this adaptive programme (PUFA sequestration into LD-stored TGs, desaturase suppression and GPX4 upregulation) the PUFA stress response, a coordinated, multilayered defence whose individual components have been described in cancer and other contexts but whose integration as a response to OXPHOS deficiency is, to our knowledge, novel.

### PUFA stress response protects the cells from lipid peroxidation

To examine the relevance of PUFA stress response in protecting against lipid peroxidation, we induced lipid peroxidation with the ferroptosis activator RSL3 (Cheff et al, 2023) and assessed lipid peroxidation with BODIPY 581/591 C11. Peroxidation was significantly higher in 6BKO than in WT after 24 h (Fig. 3D), but not elevated in 6BAOX, likely due to the sustained high GPX4 expression (Fig. 3C). Lipid peroxidation was abolished by ferrostatin-1 (the lipophilic ferroptosis inhibitor), confirming ferroptosis dependence. Consistent with prior reports (Magtanong et al, 2019), pretreatment with BSA-conjugated oleic acid (monounsaturated fatty acid) suppressed RSL3-induced lipid peroxidation across all cell lines, with a significantly greater effect in 6BKO cells than in WT (Fig. 3D). Inhibition of TG synthesis with inhibitors of diacylglycerol acyltransferases 1 and 2 (DGAT1/2) (Fig. EV2E) reduced LD formation (Fig. EV2F) without an increase in lipid peroxidation (Fig. 3D).

Relative proliferation in cells treated with DGAT1/2 inhibitors over 24 h (Fig. EV2G) or 48 h (Fig. 3E) was modestly but significantly reduced in 6BKO relative to WT. RSL3 suppressed proliferation across all cell lines, with 6BKO being most sensitive at 48 h, and combined DGAT1/2 inhibition and RSL3 did not cause a further reduction. Ferrostatin-1 fully rescued RSL3-induced growth inhibition (Figs. 3F and EV2H), and ferrostatin-1 alone modestly increased 6BKO proliferation at 48 h (Fig. 3F), suggesting a baseline ferroptotic burden. This protection was attenuated when TG synthesis was inhibited by DGAT1/2.

BSA-conjugated oleic acid (OA, 18:1) fully blocked RSL3-induced lipid peroxidation at 24 h (Fig. EV2I) or 48 h (Fig. 3G), whereas palmitic acid (PA, 16:0) did not rescue RSL3-treated 6BKO proliferation. Instead, PA further reduced proliferation in WT and 6BAOX cells (Fig. 3G), consistent with the lipotoxic effects of saturated fatty acids. Together, these results indicate that the PUFA stress response is protective. GPX4 upregulation provides a primary defence by detoxifying phospholipid hydroperoxides in situ, while TG synthesis and LD biogenesis provide an additional layer relevant to cell proliferation, even though DGAT1/2 inhibition alone does not raise lipid peroxidation.

As a proof-of-concept, re-expression of COX6B1 in 6BKO cells (6BRES, Appendix Fig. S3A) reduced TGs to WT values (Appendix Fig. S3B), and normalised or even reduced levels of highly unsaturated TG species (Appendix Fig. S3C). Fatty acid desaturase expression was normalised, but GPX4 remained elevated (as in 6BAOX cells), and iron metabolism enzymes were altered (Appendix Fig. S3D,E). RSL3-induced peroxidation tended to be lower in 6BRES than in WT cells (Appendix Fig. S3F), and proliferation under RSL3 was comparable at 24 h but improved at 48 h (Appendix Fig. S3G), likely reflecting persistent GPX4 upregulation, rather than COX6B1 re-expression per se.

The persistence of GPX4 in both 6BAOX and 6BRES after the redox trigger is removed indicates a stable transcriptional adaptation. In a cardiac CIV-deficiency model, GPX4 was induced via Nrf2- and Atf4-mediated transcription (Ahola et al, 2022) and we previously found elevated Atf4 in 6BKO (Cunatova et al, 2024), suggesting a common axis linking mitochondrial stress sensing to GPX4 induction.

### PUFA stress response is a general phenomenon in OXPHOS-limited cells

**Figure EV3:**
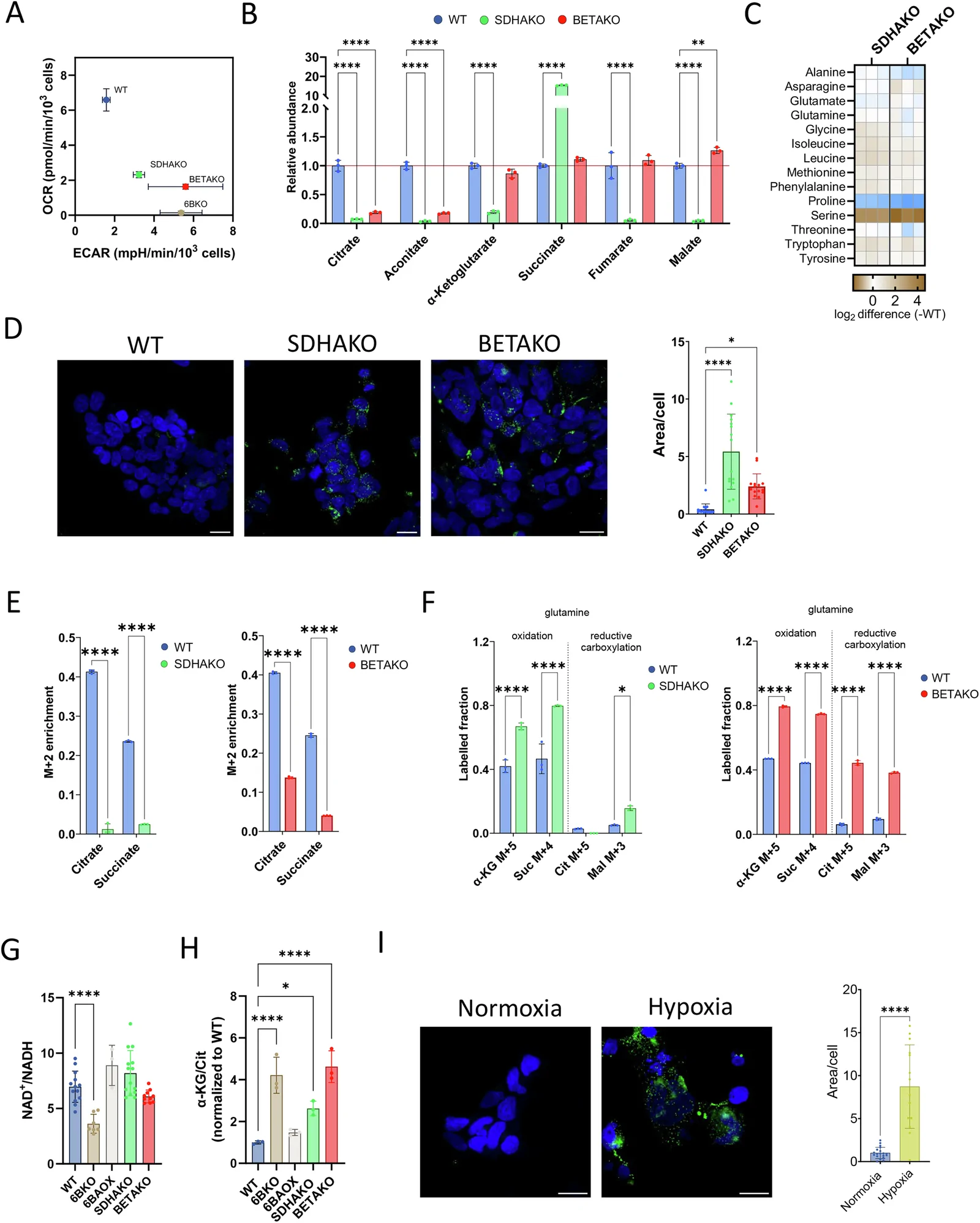
Metabolic remodelling in OXPHOS-limited cells. (**A**) Metabolic phenotype of WT, 6BKO, BETAKO (data published in (Cunatova et al, 2024)) and SDHAKO cells represented as a phenogram – combined parallel evaluation of cellular oxygen consumption rate (OCR) and extracellular acidification rate (ECAR). Data were expressed as means ± SEM (*n* ≥ 3). (**B**) Metabolic profiling (LC–MS) of TCA cycle metabolites after 24 h incubation in fresh culture media. Data are expressed as fold changes compared to WT (*n* = 3). (**C**) Heatmap showing differences in amino acid levels in SDHAKO and BETAKO cells after 24 h of incubation in culture media. Data represent log_2_(fold change) compared to WT (*n* = 3). (**D**) Representative image and quantification of BODIPY 493/503 staining of the WT, SDHAKO and BETAKO cell lines (*n* ≥ 15). Scale bar size is 20 µm. (**E**,** F**) The WT, SDHAKO and BETAKO cells were incubated with the tracer (^13^C_6_-glucose or ^13^C_5_-glutamine) for 24 h, and the labelled fraction (proportion of total pool) of TCA cycle metabolites derived from ^13^C_6_-glucose (**E**) or ^13^C_5_-glutamine (**F**) was assessed by LC–MS (*n* = 3). (**G**) NAD^+^/NADH ratio assessed in WT, 6BKO, 6BAOX, BETAKO (data published in (Cunatova et al, 2024)) and SDHAKO (*n* ≥ 3). (**H**) α-ketoglutarate/citrate (α-KG/Cit) ratio measured by LC–MS after 24 h incubation in fresh culture media. Data were expressed as fold changes compared to WT (*n* = 3). (**I**) Representative image and quantification of BODIPY 493/503 staining of the WT in normoxia or hypoxia (*n* ≥ 15). Scale bar size is 20 µm. *N* represents the number of biological replicates. Bar graphs represent means ± SD. Two-way (**B**, **E**, **F**), one-way (**D**, **G**) ANOVA or *t*-test (**I**) was performed (**B** – p** = 0.0015; **D** – p* = 0.0162; **F** – p* = 0.0115; **H** – p* = 0.0139; p**** < 0.0001).

**Figure 4:**
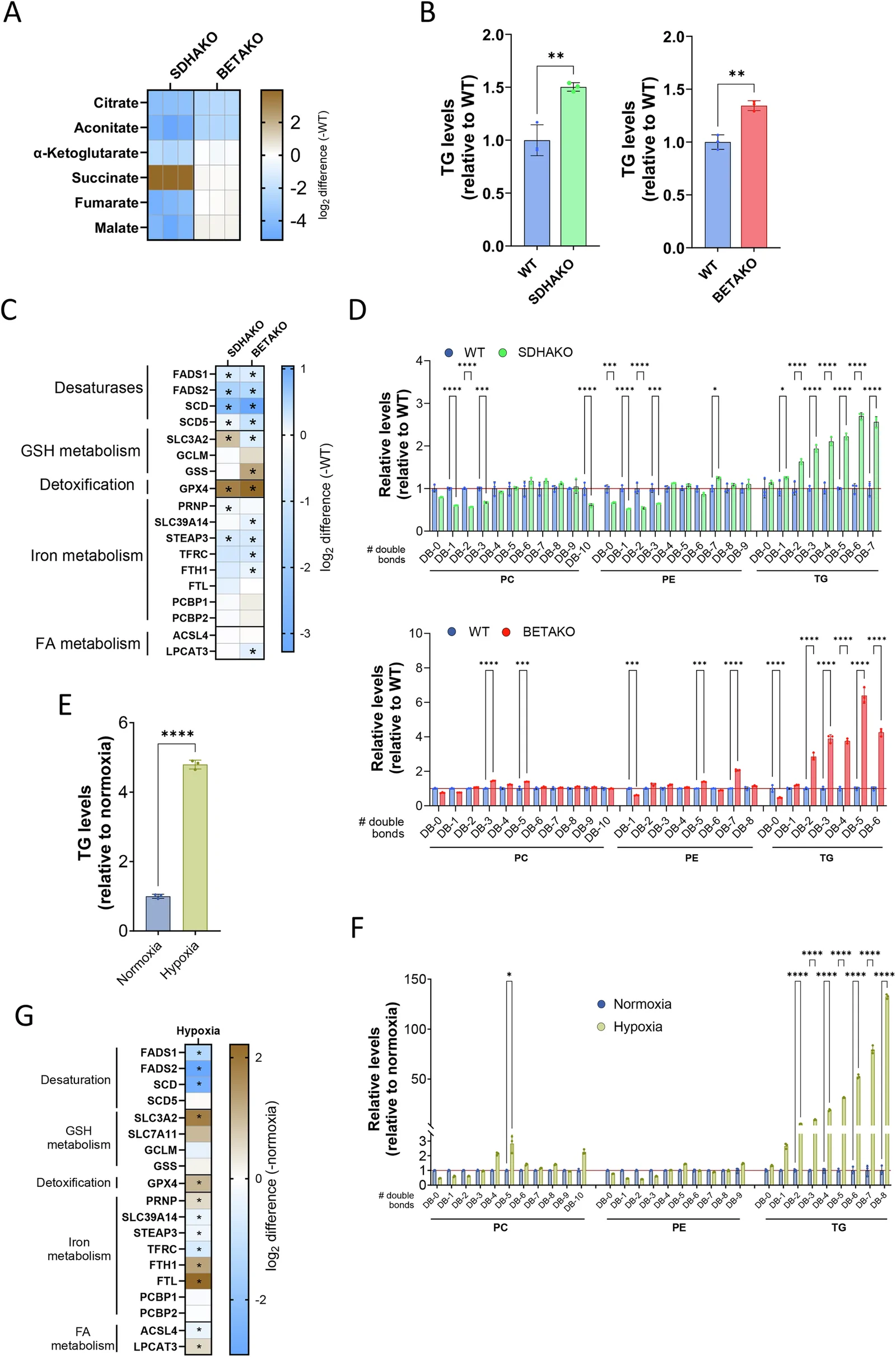
PUFA stress response is activated by OXPHOS limitation. (**A**) Heatmap showing fold changes in TCA cycle metabolite levels in SDHAKO or BETAKO cells after 24 h incubation in culture media. Data represent log_2_(fold change) compared to WT (*n* = 3). (**B**) Relative levels of TG measured by LC–MS in SDHAKO and BETAKO. (**C**) LFQ-MS of ferroptosis-related protein levels. The heatmap shows log_2_(fold change) for SDHAKO or BETAKO relative to WT (*n* = 3). Asterisks denote proteins that have changed significantly (*p* value <0.05). (**D**) Relative levels of phospholipids (PC, PE) and TG in SDHAKO and BETAKO measured by LC–MS differentiated based on double bond content (*n* = 3). (**E**) Relative levels of TG measured by LC–MS in WT exposed for 1 week to hypoxia (1% O_2_). Data were expressed as fold changes relative to normoxia (21% O_2_), *n* = 3. (**F**) Relative levels of phospholipids (PC, PE) and TG in WT exposed to hypoxia. Data were expressed as fold changes relative to normoxia (*n* = 3). (**G**) LFQ-MS analysis of ferroptosis-related protein levels. The heatmap displays log_2_(fold change) values for hypoxia relative to normoxia (*n* = 3). Asterisks denote proteins that have changed significantly (*p* value <0.05). *N* represents the number of biological replicates. Bar graphs represent means ± SD. Unpaired *t*-test (**B**, **E**) or two-way ANOVA (**D**, **F**) were performed (**B** – p** = 0.0046 (SDHAKO), 0.0020 (BETAKO); **D** – p* = 0.0353 (PE DB-7), 0.0273 (TG DB-1), p*** = 0.0008 (SDHAKO PC DB-3), 0.0007 (SDHAKO PE DB-0), 0.0002 (SDHAKO PE DB-3, BETAKO PE DB-5), 0.0001 (BETAKO PC DB-5, PE DB-1); **F** – p* = 0.0296; p**** <0.0001). Source data are available online for this figure.

To investigate the generality of the PUFA stress response, we studied HEK293 cell knockouts of the complex II (CII) subunit SDHA (SDHAKO) and the complex V (CV) subunit ATP5F1B (BETAKO), which lack the assembled complex and exhibit a secondary CI deficiency (Cunatova et al, 2024). All knockouts respired significantly less than WT, and all showed elevated glycolytic activity, as judged from extracellular acidification rate (ECAR) (Fig. EV3A). In SDHAKO cells, succinate accumulated, whereas other TCA metabolites decreased, and in BETAKO, only citrate and aconitate decreased (Fig. 4A and EV3B). In both knockouts, the amino acid profile mirrored that of 6BKO cells, with a prominent increase in serine (Fig. EV3C). Lipid profile also recapitulated the features observed in 6BKO: accumulated TGs (Fig. 4B) were stored in lipid droplets (Fig. EV3D), cellular desaturases were suppressed, and GPX4 was elevated (Fig. 4C). PUFAs were enriched in TGs more than in phospholipids (Fig. 4D; Appendix Fig. S2B,C).

Glucose and glutamine tracing (Fig. EV3E,F) showed that glucose flux through the TCA cycle was decreased in BETAKO and was negligible in SDHAKO (Fig. EV3E), and α-KG M + 5 and succinate M + 4 derived from glutamine oxidation were accumulated in both models. BETAKO resembled 6BKO, with increased reductive carboxylation (elevated citrate M + 5 and malate M + 3) and preferential use of glutamine-derived acetyl-CoA for palmitic acid synthesis (Appendix Fig. S4B). SDHAKO cells showed only a slight increase in reductive carboxylation (Fig. EV3F; Appendix Fig. S4A). Of the two proposed regulators of reductive metabolism (Fendt et al, 2013; Mullen et al, 2011), the NAD^+^/NADH ratio (Fig. EV3G) was reduced only in 6BKO cells and normalised by AOX, whereas the α-KG/citrate ratio (Fig. EV3H) was most increased in 6BKO and BETAKO (more than fourfold), indicating that a high α-KG/citrate ratio is a driver of reductive glutamine utilisation in OXPHOS-deficient cells. OXPHOS disorders generally cause redox imbalance, such as decreased NAD^+^/NADH or increased mitochondrial ROS (Goncalves et al, 2021; Mattiazzi et al, 2004; Mracek et al, 2006) that ultimately drives lipid peroxidation (Daiber, 2010). In this regard, the SDHAKO result is mechanistically informative, as a CII block leads to a distinct redox phenotype compared with CIV or CV deficiency. SDHAKO still displays the full PUFA stress signature despite an unchanged NAD^+^/NADH ratio and only marginally increased reductive carboxylation. The PUFA stress response is therefore not contingent on NADH overreduction, and the impaired electron flux appears to be a trigger per se.

To assess the physiological relevance of the response, we cultured WT cells under hypoxia (1% O_2_) for one week, a condition previously linked to OXPHOS inefficiency and lipid droplet formation (Ackerman et al, 2018; Lee et al, 2013). Hypoxia increased TGs (Fig. 4E) and lipid droplet area (Fig. EV3I). PUFAs were enriched in TGs and less in phospholipids (Fig. 4F). Desaturases (FADS1 and 2, SCD) were reduced, and GPX4 was elevated (Fig. 4G). The PUFA stress response is therefore activated whenever OXPHOS is limited either by deficiencies in OXPHOS complexes or by low oxygen. The sequestration of PUFAs into LD-stored TGs represents a common feature, and de novo synthesis of FAs is not a prerequisite for PUFA stress activation.

These findings establish LD-mediated PUFA redistribution as a central nexus in the cellular response to metabolic perturbation. It frames the LD as a redox-regulating organelle, as described in breast cancer (Jarc et al, 2018; Kump et al, 2026), acidic tumour environments (Dierge et al, 2021), *Drosophila* neuronal development (Bailey et al, 2015) and cell-cycle arrest (Lee et al, 2024). The recent reverse process described by Sokol et al. (Sokol et al, 2025), in which lipid-starved cancer cells liberate PUFAs from TGs into membranes, thereby increasing ferroptosis sensitivity, underscores that LD-membrane PUFA trafficking is deregulated and its direction is determined by cellular metabolic status. Notably, sonlicromanol, a redox modulator that attenuates lipid peroxidation and has recently received Orphan Drug Designation for OXPHOS defects (Beyrath et al, 2018; Smeitink et al, 2025), independently implicates this pathway as therapeutically relevant.

### PUFA stress response is an adaptive mechanism in patients with mitochondrial deficiency

**Figure 5:**
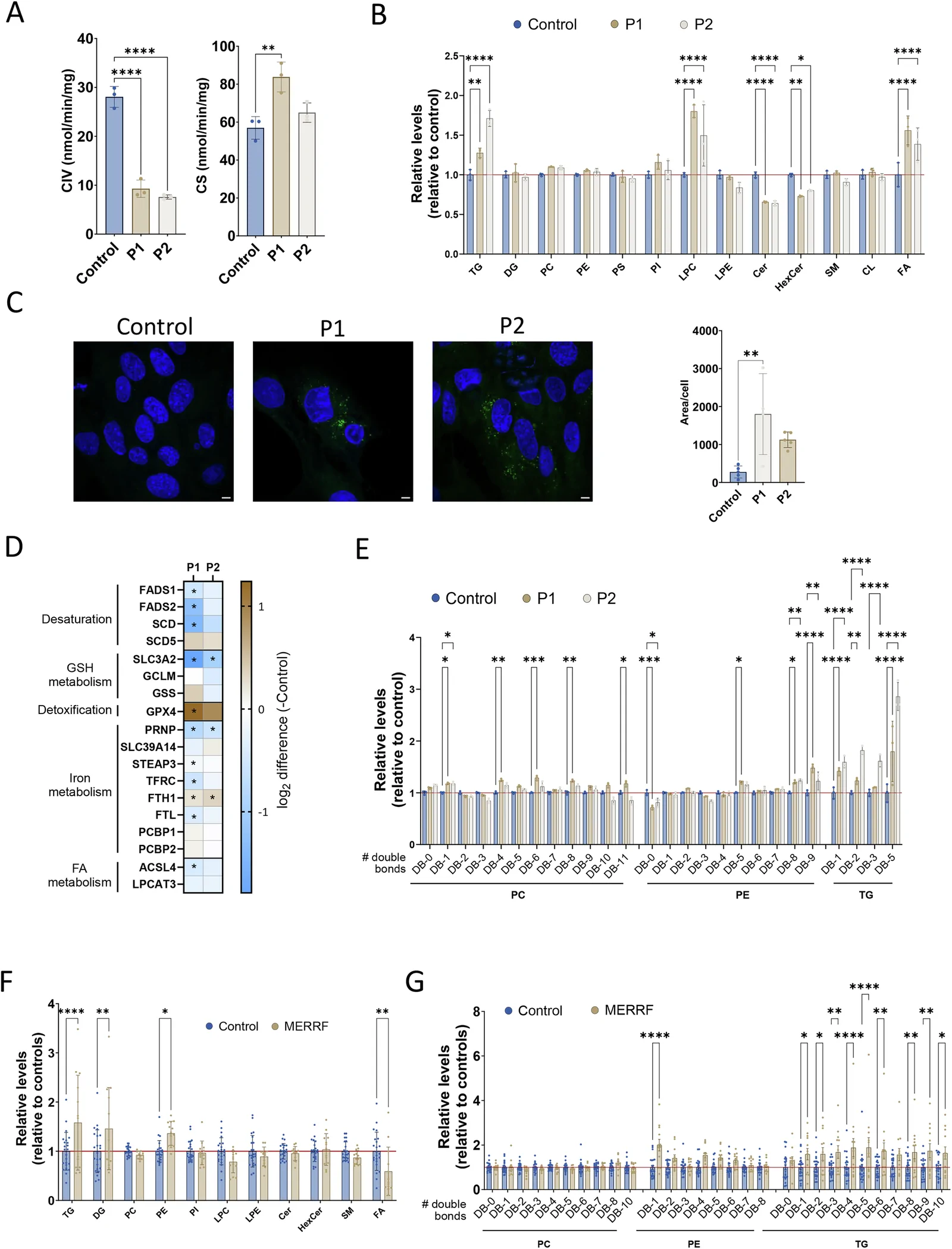
PUFA stress response in patients with mitochondrial deficiency. (**A**) Cytochrome *c* oxidase (CIV) and citrate synthase (CS) enzyme activities measured in immortalised fibroblasts (control and derived from patients with the mutation in the *COX6B1* gene – P1, P2) were assessed by spectrophotometer (*n* = 3). (**B**) Relative levels of lipid classes measured by LC–MS (labelled as in Fig. 2B). Data were expressed as fold change relative to WT (*n *= 3). (**C**) Representative image and quantification of BODIPY 493/503 staining of the control and the patients’ cells (*n* ≥ 4). Scale bar size is 100 µm. (**D**) LFQ-MS analysis of ferroptosis-related protein levels. The heatmap displays log_2_(fold change) values for P1 or P2 relative to the control (*n* = 3); asterisks denote proteins that have changed significantly (*p* value <0.05). (**E**) Relative PC, PE and TG levels differentiated based on the double bond content measured by LC–MS in control and patients’ fibroblasts (*n* = 3). Relative levels of lipid classes (**F**) and PC, PE and TG differentiated based on the content of double bonds (**G**) measured by LC–MS in plasma of controls (*n* = 23) and MERRF patients (*n* = 13). Data were expressed as fold changes relative to control. *N* represents the number of biological replicates. Bar graphs represent means ± SD. One-way (**A**, **C**) or two-way (**B**, **E**–**G**) ANOVA was performed (**A** – p** = 0.0039; **B** – p* = 0.0230, p** = 0.0011 (TG), 0.0014 (HexCer); **C** – p** = 0.0044; **E** - see Table EV3; **F** – p* = 0.0308, p** = 0.0020 (DG), 0.0093 (FA); **G** – *p*- = 0.0362 (DB-1), 0.0282 (DB-2), 0.0134 (DB-10), p** = 0.0049 (DB-2), 0.0016 (DB-6), 0.0053 (DB-8), 0.0014 (DB-9); p****  < 0.0001). Source data are available online for this figure.

Next, we investigated whether PUFA stress response is relevant to human pathology. Immortalised human skin fibroblasts from two patients carrying the COX6B1 p.R20H variant (Massa et al, 2008) had significantly decreased cytochrome *c* oxidase activity (Fig. 5A), increased TG and free FA levels (Fig. 5B), and accumulated LDs (Fig. 5C). Like the knockout models, patient cells displayed reduced desaturases (SCD, FADS1 and FADS2), elevated GPX4 (Fig. 5D), and highly elevated PUFAs in TGs and less in phospholipids (Fig. 5E).

To determine the relevance of the response in vivo, we compared plasma samples from controls and patients with myoclonic epilepsy with ragged-red fibres (MERRF) syndrome (Antonicka et al, 1999). In accordance with a previous study (Vankoningsloo et al, 2005), the patients’ plasma showed increased TGs and DGs (Fig. 5F), indicating fatty acid overload or upregulated lipid synthesis. The rise of PE may reflect altered turnover, e.g. during mitophagy, given the high PE content in mitochondrial membranes (Decker and Funai, 2024). PUFA-enriched circulating TGs were accumulated in MERRF patients (Fig. 5G). Together, the patient fibroblasts and MERRF plasma data indicate that the PUFA stress response operates in vivo in human mitochondrial disease.

Given the documented plasma lipid imbalance in patients with mitochondrial disease (Ruiz et al, 2019), elevated plasma PUFA-TGs could serve as a disease-relevant biomarker, although our cohort (13 MERRF patients, 23 controls) is exploratory and requires validation in larger, genetically diverse datasets. These findings also carry therapeutic implications: DGAT inhibitors that are in clinical trials for metabolic dysfunction-associated steatotic liver disease (Longo et al, 2024) could block PUFA sequestration and unmask ferroptosis sensitivity in OXPHOS-limited tissues. Conversely, a MUFA-enriched diet might reduce ferroptosis risk as the oleic acid abrogated RSL3-induced peroxidation and restored proliferation in all lines (Fig. 3D,G) (Magtanong et al, 2019), though hepatic lipid accumulation must be weighed (Longo et al, 2024). Rational exploitation of the PUFA stress response will require dissecting the upstream signals that coordinate its three components.

## Methods

**Table:** Reagents and tools table

Reagent/resource	Reference or source	Identifier or catalogue number
**Experimental models**		
Human: HEK293 cell line (WT)	ATCC	CRL-1573
COX6B1 KO HEK293 cell line (6BKO)	Cunatova et al, 2024	N/A
6BKO with stable AOX xeno-expression 6BKO (6BAOX)	Cunatova et al, 2024	N/A
6BKO with stable COX6B1 expression (6BRES)	Cunatova et al, 2026	N/A
SDHAKO HEK293 cell line (SDHAKO)	Cunatova et al, 2024	N/A
ATP5F1B KO HEK293 cell line (BETAKO)	Kovalcikova et al,	N/A
Immortalised human fibroblasts derived from patients with the *COX6B1* mutation (c.221 G > A)	Massa et al, 2008	N/A
**Recombinant DNA**		
N/A		
**Antibodies**		
GPX4	Abcam	ab125066
hnRNP E1	Cell Signalling	8534S
COX6B1	Proteintech	11425-1-AP
Donkey anti-Rabbit IgG (H + L) Highly Cross-Adsorbed	Thermo Fisher	A10043
**Oligonucleotides and other sequence-based reagents**		
N/A		
**Chemicals, enzymes and other reagents**		
DMEM/F-12 medium	Biowest	L0092
DMEM/F-12 medium (no glutamine, glucose and HEPES)	Biowest	L0091
DMEM	VWR	392-0415
DMEM (Seahorse)	Merck	D5030
FluoroBrite™ DMEM	Thermo Fisher Scientific	A1896701
L-glutamine	Thermo Fisher Scientific	25030-029
Glucose solution	Thermo Fisher Scientific	A24940-01
Penicillin-Streptomycin	Thermo Fisher Scientific	15140-122
FBS	Thermo Fisher Scientific	10270-106
Dialysed FBS	Thermo Fisher Scientific	26400044
^13^C_6_-glucose	Cambridge Isotope Laboratories	CLM-3612-1
^13^C_5_–glutamine	Cambridge Isotope Laboratories	CLM-1822-H-0.1
DGAT1 inhibitor (A922500)	Merck	A1737
DGAT2 inhibitor (PF-06424439)	Merck	PZ0233
SCD1 inhibitor	Cayman Chemical	CAY10566
FADS1 and FADS2 inhibitor (CP-24879)	Merck	C9115-5MG
FADS2 inhibitor (SC-26196)	Merck	PZ0176-5MG
Ferrostatin-1	Merck	SML0583
RSL3	Merck	SML2234
CyQUANT Cell Proliferation Assay	Thermo Fisher Scientific	C7026
Oleic acid	Merck	O7501
Pamitic acid	Merck	P9767
Carnitine	Merck	C0283
Etomoxir	Merck	E1905
[9,10(n)-3H] palmitic acid	Revvity	NET043001MC
Formaldehyde solution	Merck	F1635
BODIPY^TM^ 581/591 C11	Thermo Fisher Scientific	D3861
BODIPY^TM^ 558/568	Thermo Fisher Scientific	D3835
DAPI	Thermo Fisher Scientific	D1306
Cytochrome c	Merck	C2037
Acetyl coenzyme A	Merck	A2056
Oxaloacetic acid	Merck	O4126
5,5′-Dithiobis(2-nitrobenzoic acid)	Merck	D8130
Poly-L-lysine solution	Merck	P8920
Sodium Pyruvate	Merck	P2256
Oligomycin	Merck	O4876
Carbonyl cyanide 4-(trifluoromethoxy)phenylhydrazone (FCCP)	Merck	C2920
Rotenone	Merck	R8875
Antimycin A	Merck	A8674
2-deoxy-D-glucose	Merck	D8375
Hoechst 33342	Thermo Fisher Scientific	H1399
Pierce blocking buffer	Thermo Fisher Scientific	37570
NAD + /NADH Glo assay	Promega	G9071
GSH/GSSG Glo assay	Promega	V6611
**Software**		
GraphPad Prism 10	GraphPad software	
Gen5 software	BioTek	
FlowJo 10	BD Biosciences	
FIJI ImageJ	Schindelin et al, 2012	
Wave	Agilent	
Spectronaut 19.9	Biognosys	
Perseus (v. 2.1.3.0)	Tyanova et al, 2016	
BioRender	BioRender.com	
**Other**		
N/A		

### Human cell lines and primary fibroblasts

COX6B1 (6BKO), SDHA (SDHAKO) and ATP5F1B (BETAKO) HEK293 (ATCC CRL-1573) knock-out cells were previously generated (Cunatova et al, 2024; Kovalcikova et al, 2019). For alternative oxidase expression in 6BKO cells (to produce 6BAOX), pcDNA3.1+ mammalian expression vector (Thermo Fisher Scientific) containing the coding sequence of full-length AOX (from *Aspergillus nidulans)*, followed by a C-terminal HA-tag, was transfected as described in (Cunatova et al, 2024). Stably transfected cells were selected with 2 mg/mL G418. The HEK-derived cell lines were maintained at 37 °C and 5% CO_2_ atmosphere in DMEM/F-12 medium (Biowest, L0092) supplemented with 10% (v/v) FBS (Thermo Fisher Scientific), 40 mM HEPES, antibiotics (100 U/mL penicillin + 100 μg/mL streptomycin, Thermo Fisher Scientific) and 50 µM uridine. To assess the impact of hypoxia, the WT cells were cultured in normoxia (21% O_2_) or placed in a hypoxic chamber (1% O_2_, Xvivo System X3) for 1 week. Label-free quantification mass spectrometry samples, lipid droplets quantification and metabolomic and lipidomic analyses were prepared as described below in a hypoxic chamber.

Fibroblasts from the patients and controls were immortalised as described previously (Massa et al, 2008). The cells were cultured in Dulbecco’s modified Eagle’s medium (VWR) supplemented with 10% (v/v) FBS (Thermo Fisher Scientific), 20 mM HEPES and antibiotics (100 U/mL penicillin + 100 μg/mL streptomycin, Thermo Fisher Scientific).

### Human plasma

Following an overnight fast, blood samples were drawn from the antecubital vein of patients and healthy controls between 7:00 and 8:00 a.m. Plasma was collected by centrifugation (2000 RPM, RT, 10 min) from one mL EDTA-treated blood and aliquots were stored at −80 °C until lipidomic analyses. A total of 13 patients with MERRF syndrome (13–56 years old, 69% females, 31% males) and 23 age-matched healthy controls (17–80 years old, 70% females, 30% males) were included. The study was performed in accordance with The Code of Ethics of the World Medical Association (Declaration of Helsinki), and the study protocol was approved by the Ethical Review Board of the First Faculty of Medicine and General University Hospital in Prague (77/21 Grant AZV VES 2022 VFN, 17.6.2021). Written informed consent was obtained from all subjects.

### Sample preparation and extraction for LC–MS analysis

#### Sample preparation

Cells were seeded in triplicate in a six-well plate (4.5–6 × 10^5^ cells/well) and cultured in growth media (see above) for 24 h. For profiling, 200 µl of fresh and used medium was frozen immediately in liquid nitrogen. For fluxomics, the cultured medium was replaced with DMEM/F-12 medium without glutamine, glucose and HEPES (Biowest, L0091) supplemented with 10% (v/v) dialysed FBS (Thermo Fisher Scientific), 2 mM L-glutamine (Thermo Fisher Scientific), 10 mM glucose (Thermo Fisher Scientific), 40 mM HEPES, antibiotics (100 U/mL penicillin + 100 μg/mL streptomycin, Thermo Fisher Scientific) and 50 μM uridine. After 2 h, the medium was changed for tracing medium – DMEM/F-12 medium (Biowest, L0091) supplemented with 10% (v/v) dialysed FBS (Thermo Fisher Scientific), 40 mM HEPES, antibiotics (100 U/mL penicillin + 100 μg/mL streptomycin, Thermo Fisher Scientific), and 50 μM uridine. The glucose tracing medium was further supplemented with 2 mM L-glutamine and 10 mM ^13^C_6_-glucose (CIL). The glutamine tracing medium with 2 mM ^13^C_5_-glutamine (CIL) and 10 mM glucose. The cells were incubated in tracing medium for 24 h to reach pseudo-steady-state labelling conditions, and data are expressed as a proportion of the total pool (fractional labelling) to account for differences in cell proliferation rates. The cells were then rinsed twice in ice-cold PBS, the medium was aspirated, and the cells were immediately frozen at -80 °C.

#### Cell extraction

A volume of 275 µL of methanol and 275 µL of 10% methanol containing internal standards was added directly to the cells in a six-well plate, and the cells were scraped off (Cajka et al, 2023). The suspension was transferred to a 2-mL Eppendorf tube containing a milling ball. After homogenisation (1 min), 1 mL of MTBE containing internal standards was added. The tubes were vortexed (20 s) and centrifuged (16,000 rpm, 5 min, 4 °C).

For metabolomics, a 70 µL aliquot of the bottom phase was collected and evaporated to dryness. The dry extracts were resuspended in 70 µL of acetonitrile/water (4:1) containing two internal standards (12-[[(cyclohexylamino)carbonyl]amino]-dodecanoic acid (CUDA) and Val-Tyr-Val), shaken (30 s), centrifuged (16,000 rpm, 5 min, 4 °C), and analysed using HILIC metabolomics platforms (Cajka et al, 2023).

Another 70 µL aliquot of the bottom phase was mixed with 210 µL of isopropanol/acetonitrile (1:1), shaken (30 s), centrifuged (16,000 rpm, 5 min, 4 °C), and the supernatant was evaporated. The dry extracts were resuspended in 5% methanol/0.2% formic acid containing two internal standards (CUDA and Val-Tyr-Val), shaken (30 s), centrifuged (16,000 rpm, 5 min, 4 °C), and analysed using the RPLC metabolomics platform (Cajka et al, 2023).

For lipidomics, a 300 µL aliquot of the upper phase was collected, evaporated to dryness, and the extracts were resuspended in 100 µL of methanol containing the internal standard CUDA, shaken (30 s), centrifuged (16,000 rpm, 5 min, 4 °C), and analysed using the RPLC lipidomics platform (Cajka et al, 2023).

For fatty acid profiling, a 300 µL aliquot of the upper phase was collected, evaporated to dryness, and dissolved in 500 µL of 0.3 M KOH in methanol/water (9:1), followed by vortexing (10 s). The mixture was heated at 55 °C for 1 h while shaking (200 rpm). Subsequently, 50 µL of 3 M HCl in methanol was added, and the mixture was vortexed. If necessary, the pH was further adjusted to neutral by adding small volumes of 3 M HCl in methanol, followed by the addition of 500 µL of water. Free fatty acids were extracted with 500 µL of hexane, vortexed (30 s), and centrifuged (2000×*g*, 5 min, 4 °C). An aliquot of 200 µL of the hexane layer was transferred to a tube and evaporated to dryness. The residues were dissolved in 100 µL of methanol containing the internal standard CUDA, vortexed (10 s), centrifuged (16,000 rpm, 5 min, 4 °C), and transferred to a vial.

#### Medium extraction

A volume of 175 µL of medium was mixed with 180 µL of methanol and shaken (30 s). Subsequently, 600 µL of MTBE was added, the mixture was vortexed (10 s), and centrifuged (16,000 rpm, 5 min, 4 °C).

For metabolomics, the same 70 µL aliquots of the bottom phase were collected as described for the cells, but resuspended in 490 µL of solvents compatible with the HILIC and RPLC platforms.

For lipidomics, an aliquot of 250 µL of the upper phase was collected, evaporated to dryness, and resuspended in 50 µL of methanol containing the CUDA internal standard.

### LC–MS analysis

The LC–MS systems consisted of a Vanquish UHPLC system (Thermo Fisher Scientific), an OptaMax NG ion source with a HESI-II probe (Thermo Fisher Scientific), and an Orbitrap Exploris 480 mass spectrometer (Thermo Fisher Scientific). Samples underwent biphasic extraction to separate polar metabolites and complex lipids, which were analysed using a multiplatform LC–MS approach. Profiling methods are based on acquisition of MS1 spectra with data-dependent acquisition (DDA) MS/MS for metabolite annotation, whereas fluxomic methods rely on acquisition of high-resolution MS1 spectra (180,000 FWHM).

#### Metabolomics

The ACQUITY Premier BEH Amide column (50 mm × 2.1 mm i.d.; 1.7-μm particle size) with a VanGuard FIT cartridge (5 mm × 2.1 mm i.d.; 1.7-μm particle size) (Waters, Milford, MA, USA) was used to separate polar metabolites based on the HILIC mechanism. The column was maintained at 45 °C at a flow rate of 0.4 mL/min. The mobile phases were (A) water containing 10 mM ammonium formate and 0.125% formic acid, and (B) acetonitrile/water (95:5) containing 10 mM ammonium formate and 0.125% formic acid. Separation was conducted under the following gradient: 0 min 100% (B); 0–1 min 100% (B); 1–3.9 min from 100% to 70% (B); 3.9–5.1 min from 70% to 30% (B); 5.1–6.4 min from 30% to 100% (B); 6.4–7.5 min 100% (B) + 1 min preinjection steps. An injection volume of 1 μL was used. The sample temperature was maintained at 4 °C. The ESI source parameters were: sheath gas pressure, 50 arbitrary units; aux gas flow, 13 arbitrary units; sweep gas flow, 3 arbitrary units; capillary temperature, 260 °C; aux gas heater temperature, 425 °C; spray voltage, +3.6 kV. For metabolomic profiling, the MS settings were: MS1 mass range, m/z 60–900; MS1 resolving power, 45,000 FWHM; the number of data-dependent scans per cycle, 5; MS/MS resolving power, 15000 FWHM; normalised collision energies, 30, 40 and 50% (Benova et al, 2023; Cajka et al, 2023). For fluxomic analysis, the MS settings were: MS1 mass range, m/z 60–900; MS1 resolving power, 180,000 FWHM.

The ACQUITY Premier HSS T3 column (50 mm × 2.1 mm i.d.; 1.8-μm particle size) with a VanGuard FIT cartridge (5 mm × 2.1 mm i.d.; 1.8-μm particle size) (Waters) was used to separate polar metabolites based on the RPLC mechanism. The mobile phases were (A) water containing 0.2% formic acid and (B) methanol containing 0.1% formic acid. Separation was conducted under the following gradient: 0 min 1% (B) 0.3 mL/min; 0–0.5 min 1% (B) 0.3 mL/min; 0.5–2 min from 1% to 60% (B) 0.3 mL/min; 2–2.3 min from 60% to 95% (B) from 0.3 mL/min to 0.5 mL/min; 2.3–3.0 min 95% (B) 0.5 mL/min; 3.0–3.1 min from 95% to 1% (B) 0.5 mL/min; 3.1–4 min 1% (B) 0.5 mL/min; 4–4.1 min 1% (B) from 0.5 mL/min to 0.3 mL/min; 4.1–4.5 min 1% (B) 0.3 mL/min + 1 min preinjection steps. An injection volume of 5 μL was used. The sample temperature was maintained at 4 °C. The ESI source parameters were: sheath gas pressure, 50 arbitrary units; aux gas flow, 13 arbitrary units; sweep gas flow, 3 arbitrary units; capillary temperature, 260 °C; aux gas heater temperature, 425 °C; spray voltage, −2.5 kV. For metabolomic profiling, the MS settings were: MS1 mass range, m/z 60–900; MS1 resolving power, 45,000 FWHM; the number of data-dependent scans per cycle, 5; MS/MS resolving power, 15,000 FWHM; normalised collision energies, 30, 40 and 50% (Benova et al, 2023; Cajka et al, 2023). For fluxomic analysis, the MS settings were: MS1 mass range, m/z 60–900; MS1 resolving power, 180,000 FWHM.

#### Lipidomics

The ACQUITY Premier BEH C18 column (50 mm × 2.1 mm i.d.; 1.7-μm particle size) equipped with a VanGuard FIT cartridge (5 mm × 2.1 mm i.d.; 1.7-μm particle size) (Waters) was utilised to separate complex lipids based on the RPLC mechanism. The separation was carried out at a flow rate of 0.6 mL/min, with the column maintained at 65 °C. For LC-ESI(+)-MS lipidomic analysis, the mobile phases were (A) 60:40 acetonitrile/water containing 10 mM ammonium formate and 0.1% formic acid and (B) 90:10:0.1 isopropanol/acetonitrile/water containing 10 mM ammonium formate and 0.1% formic acid. For LC-ESI(−)-MS lipidomic analysis, the mobile phases were (A) 60:40 acetonitrile/water containing 10 mM ammonium acetate and 0.1% acetic acid, and (B) 90:10:0.1 isopropanol/acetonitrile/water containing 10 mM ammonium acetate and 0.1% acetic acid. Separation was conducted under the following gradient for LC-ESI(+)-MS: 0 min 15% (B); 0–1 min from 15 to 30% (B); 1–1.3 min from 30% to 48% (B); 1.3–5.5 min from 48 to 82% (B); 5.5–5.8 min from 82 to 99% (B); 5.8–6 min 99% (B); 6–6.1 min from 99 to 15% (B); 6.1–7 min 15% (B) + 1 min preinjection steps. For the LC-ESI(−)-MS, the following gradient was used: 0 min 15% (B); 0–1 min from 15 to 30% (B); 1–1.3 min from 30 to 48% (B); 1.3–4.8 min from 48 to 76% (B); 4.8–4.9 min from 76 to 99% (B); 4.9–5.3 min 99% (B); 5.3–5.4 min from 99 to 15% (B); 5.4–6.3 min 15% (B) +1 min preinjection steps. Injection volumes of 1 and 5 μL were used for ESI(+) and ESI(–) analysis, respectively. The sample temperature was maintained at 4 °C. The ESI source parameters were: sheath gas pressure, 60 arbitrary units; aux gas flow, 25 arbitrary units; sweep gas flow, 2 arbitrary units; capillary temperature, 250 °C; aux gas heater temperature, 250 °C; spray voltage, +3.6 kV for ESI(+), spray voltage, −3.0 kV for ESI(–). For lipidomic profiling, the MS settings were: MS1 mass range, m/z 200–1700; MS1 resolving power, 45,000 FWHM; the number of data-dependent scans per cycle, 5; MS/MS resolving power, 15,000 FWHM; normalised collision energy, 25% for ESI(+); normalised collision energies, 20 and 30% for ESI(–) (Benova et al, 2023; Cajka et al, 2023). For fluxomic analysis, the MS settings were: MS1 mass range, m/z 200–1700; MS1 resolving power, 180,000 FWHM.

For free fatty acid profiling, the mobile phase were the same as in LC-ESI(−)-MS lipidomic profiling, but with shorter gradient: 0 min 15% (B); 0–1 min from 15 to 30% (B); 1–1.3 min from 30 to 48% (B); 1.3–3.05 min from 48 to 62% (B); 3.05–3.2 min from 76 to 99% (B); 3.2–3.6 min 99% (B); 3.6–3.7 min from 99% to 15% (B); 3.7–4.6 min 15% (B) + 1 min preinjection steps. The ESI settings were the same as for lipidomic profiling. The MS settings were: MS1 mass range, m/z 195–600; MS1 resolving power, 180,000 FWHM.

#### Quality control

Quality control included (i) randomisation of the samples within the sequence, (ii) regular injection of quality control (QC) pool samples at the beginning, end, and between every ten actual samples, (iii) analysis of method blanks, (iv) analysis of serial dilution samples prepared from QC sample (0, 1/16, 1/8, 1/4, 1/2, 1), and (v) control of the chromatographic peak shape, retention time, and intensity of internal standards (Benova et al, 2023).

#### Data processing

The LC–MS instrumental files were processed using MS-DIAL v. 4.9.221218 software (Tsugawa et al, 2020). Polar metabolites were annotated based on retention time–m/z match from an in-house spectral library, along with MS/MS libraries available from various sources (NIST23, MassBank.us, and MS-DIAL MS/MS library v. 17). Complex lipids were annotated using in silico MS/MS spectra available in MS-DIAL software. Exported datasets for each platform as signal intensity from the detector (peak heights) were filtered by removing metabolites with (i) a max sample peak height/blank peak height average <10, (ii) an R2 <0.8 from a dilution series of QC samples, and (iii) a relative standard deviation (RSD) >30% from QC samples. Data were normalised using locally estimated scatterplot smoothing (LOESS) with QC samples (Benova et al, 2023). The processed data were analysed in MetaboAnalyst 5.0 as described in (Pecina et al, 2024). Fluxomics data were processed using MRMPROBS v. 3.73.2 software to provide peak heights of ^13^C-isotopologues based on the total number of carbon atoms per analyte. Isotopic labelling was corrected for natural ^13^C-abundance using IsoCor software (Lopez-Lengowski et al, 2021).

### Lipid peroxidation analysis

The cells were seeded on a 24-well plate (2.5 × 10^5^ cells per well). After 24 h, the cells were stained with 1 µM BODIPY 581/591 C11 (Thermo Fisher Scientific) in HBSS for 30 min at 37 °C, and then the medium was replaced with culture medium containing either vehicle or 1 µM RSL3 (Merck). Where indicated, the cells were pretreated for 48 h with the mix of 1 μM DGAT1 (A922500, Merck) and 10 μM DGAT2 (PF-06424439, Merck) inhibitors (DGAT1/2i), or for 24 h with 200 nM ferroptosis inhibitor ferrostatin-1 (Merck) or 25 μM BSA-conjugated oleic acid. Following the treatment, the cells were harvested, centrifuged and resuspended in FluoroBrite medium (Thermo Fisher Scientific). The cells were analysed by flow cytometry using 488 nm emission and 530/30 nm excitation for oxidised BODIPY C11, or 561 nm emission and 615/20 nm excitation for non-oxidised BODIPY C11.

### Proliferation

Studied cell lines were seeded on a 96-well plate (5 × 10^3^ cells per well). Where indicated, the cells were pretreated for 48 h with the mix of 1 µM DGAT1 (A922500, Merck) and 10 µM DGAT2 (PF-06424439, Merck) inhibitors (DGAT1/2i), or for 24 h with 200 nM ferroptosis inhibitor ferrostatin-1 (Merck) or 25 µM BSA-conjugated palmitic or oleic acid. After 24 h, the culture medium was replaced with medium containing pretreatment and either vehicle or 1 µM RSL3 (Merck). After 24 and 48 h, the plate was dumped, blotted and stored at -80 °C. Cell proliferation was measured using the CyQUANT Cell Proliferation Assay (Thermo Fisher Scientific), following the manufacturer´s instructions. Fluorescence intensity at 480/520 nm (ex/em) was recorded using an Infinite M200 plate reader (Tecan Group Ltd., Switzerland), and growth rates were calculated as the fold change in intensity relative to the vehicle. Doubling time (DT) was calculated as: DT = (t2-t1)*log2/log(I_t2_/I_t1_), where I_t1_ and I_t2_ are fluorescence intensities at time t1 and t2 that are proportional to cell number. The data were normalised to WT values. The measurement was performed in six independent experiments.

### Fatty acid oxidation

Cells were seeded on a 24-well plate (2 × 10^5^ cells/well). After 24 h, the cultured medium was replaced with medium containing 100 μM palmitic acid (Merck), 1 mM carnitine (Merck) and 1.7 μCi [9,10(n)-^3^H] palmitic acid (GE Healthcare, UK) in the presence or absence of 100 μM etomoxir (Merck) and the cells were incubated for 4 h. Medium was collected to analyse the amount of released ^3^H_2_O that was formed during the cellular oxidation of [^3^H]-palmitate as described in (Hermanova et al, 2016). The etomoxir-sensitive portion was presented in nCi/mg protein/h. The measurement was performed in three independent experiments.

### Fatty acid conjugation

Both sodium palmitate (Merck) and oleate (Merck) were conjugated to BSA at a 1:6 ratio to the final concentration of 1 mM (Angelini et al, 2022). Briefly, fatty acids were dissolved in 150 mM NaCl at 70 °C, until no longer cloudy. Then, they were sequentially added to 34 mM BSA in 150 mM NaCl and stirred for 1 h at 37 °C. Finally, the volume was adjusted with 150 mM NaCl to yield a 1 mM final solution, and the pH was adjusted to 7.4.

### Metabolic modelling

To conduct metabolic flux analysis, the INCA software (Rahim et al, 2022) was used. A simple model, encompassing glycolysis, Krebs cycle, biosynthesis of palmitic acid from acetyl-CoA, gradual biosynthesis of stearic, oleic and palmitoleic acid and, eventually, biosynthesis of triacylglycerols (TG 50:1, TG 48:1) and phospholipid (POPC) from building blocks defined in previous steps, was defined as an approximation of relevant lipidomic pathways. MS data describing the isotopic enrichment of the fatty acids and lipid molecules mentioned above were introduced to said model, and metabolic fluxes were estimated in INCA to minimise the differences between observed and estimated isotopic enrichment values. To estimate the data published, a ratio of flux of newly synthesised palmitic acid to POPC and to all the lipids considered (POPC and triacylglycerols) was calculated.

### Lipid droplet imaging

Cells (4–8 × 10^5^) were seeded onto glass coverslips in six-well plates and incubated for 24 h. The cells were fixed in 3.7% PFA (Merck) in warm PBS for 20 min, washed twice with PBS and stored at 4 °C for a maximum of 2 weeks before imaging. Samples were incubated with BODIPY^TM^ 558/568 (1:2000, Thermo Fisher Scientific) and DAPI (5 µg/mL, Thermo Fisher Scientific) for 30 min, protected from light, followed by two 10-minute washes in PBS. Z-stack images were acquired using a Leica SP8 WLL MP laser scanning confocal microscope (HC PL APO 63x water immersion objective (NA 1.20)). The z-step size was set to 300 nm, and the confocal zoom was adjusted to maintain a pixel size of ∼100 nm.

Image analysis was performed using the FIJI/ImageJ software (Schindelin et al, 2012). For each z-stack, a maximum-intensity projection was generated, followed by the background subtraction. A consistent threshold was then applied across all images to segment BODIPY-positive signal corresponding to lipid droplets. The “Analyse Particles” function was used to measure the total BODIPY-stained area per image (µm²), which served as a proxy for total lipid droplet content. The FIJI macro used for image processing and analysis is available at GitHub. To normalise lipid droplet content for differences in cell number across images, nuclei were counted manually from maximum-intensity projections of the DAPI channel. The total BODIPY-stained area was then divided by the number of DAPI-counted nuclei in the same field of view, yielding a per-cell lipid droplet area (µm² per cell).

### Label-free quantification mass spectrometry analysis

Label-free quantification mass spectrometry analysis (LFQ-MS) of cell pellets was performed in triplicate by the Proteomics Service Laboratory at the Institute of Physiology and the Institute of Molecular Genetics of the Czech Academy of Sciences. Cellular pellets (100 µg of protein) solubilised by sodium deoxycholate (final concentration 1%) were processed according to the ethylacetate extraction in-solution trypsin digestion protocol as described in (Cunatova et al, 2021). About 500 ng of desalted peptide digests were separated on 50 cm C18 column (EASY-Spray ES903, Thermo Fisher Scientific) using 120 min elution gradient (Dionex Ultimate 3000, flow rate 300 nL/min) and acquired in a DIA mode on an Orbitrap Exploris 480 mass spectrometer (Thermo Fisher Scientific). Thermo raw files were processed by the directDIA mode and visualised in Spectronaut 19.9 (Biognosys) software using the default settings with Precursor and Protein *Q*-value and PEP cutoff set at 0.01. MMTS alkylated cysteine was selected as a fixed modification, methylthio (C). Search was performed against the human reference proteome UP000005640_9606.fasta (UniProt release 2024_01). Protein group quantities (PG.Quantity, MS2 level) from the Protein report generated by the Spectronaut were evaluated in Perseus v. 2.1.3.0 (Tyanova et al, 2016) and further visualised in GraphPad Prism.

### SDS-PAGE/WB analysis

Protein separation under denaturing conditions was performed using tricine-sodium dodecyl polyacrylamide gel electrophoresis (SDS-PAGE). Protein samples were prepared from frozen cellular pellets using RIPA buffer (150 mM NaCl, 1% Nonidet NP-40, 1% sodium deoxycholate, 0.1% SDS, 50 mM Tris, pH 8.0) as described (Cunatova et al, 2024). Proteins (35 μg) were separated on 12% polyacrylamide gels using the Mini-PROTEAN III apparatus (Bio-Rad), transferred to polyvinylidene difluoride membranes (Immobilon FL 0.45 μm; Merck) by semidry electroblotting (0.8 mA/cm^2^, 1 h) using a Transblot SD apparatus (Bio-Rad) and the membrane was blocked with Pierce blocking buffer for 1 h (Thermo Scientific). Immunodetection was performed as described (Pajuelo Reguera et al, 2020), using primary and fluorescent secondary antibodies listed in the Reagents and tools table, and was detected using the Odyssey fluorescence scanner (LI-COR Biosciences). The resulting signals were analysed and quantified by Image Lab software (Bio-Rad). Experiments were performed three times to assess the statistical significance of the results.

### Enzyme activities

Complex IV and citrate synthase (CS) activities were determined spectrophotometrically (Pecinova et al, 2011) at 30 °C in cell lysates. The complex IV assay medium consisted of 40 mM K-Pi, 1 mg/ml BSA, and a pH of 7.0. The reaction was started with 30 μM reduced cytochrome *c*, and its oxidation was monitored at 550 nm for 40 s. Citrate synthase (CS) activity was determined using a medium containing 0.1 M Tris–HCl, 0.1 mM 5,5′-dithiobis-(2-nitrobenzoic acid), 50 μM acetyl coenzyme A, pH 8.1. The reaction was initiated by adding 0.5 mM oxaloacetate and then monitored for changes at 412 nm for 1 min. The data were corrected for the absorbance change in the absence of oxaloacetate. Enzyme activities were expressed as nmol/min/mg protein using molar absorption coefficient ε_550_ = 19.6 mM/cm (complex IV) or ε_412_ = 13.6 mM/cm (CS).

### Evaluation of metabolic fluxes

Seahorse extracellular flux (XF) Analyser (Agilent Technologies, USA) was used to assess mitochondrial respiration and glycolytic rate in parallel, as described before (Cunatova et al, 2024). Briefly, 2–3.5 × 10^4^ cells were seeded on poly-L-lysine-coated 24-well measuring plate and incubated overnight under standard conditions. Before the evaluation, the cells were rinsed with 1 mL of measuring media (DMEM (Merck, D5030; pH 7.4, 37 °C) supplemented with 0.2% (w/w) BSA and 1 mM pyruvate). Cells were incubated in measuring medium for 30 min at 37 °C. The oxygen consumption rate (OCR) and extracellular acidification rate (ECAR) were recorded at basal metabolic rate and after subsequent additions of 10 mM glucose, 1 µM oligomycin, 1 µM FCCP, and a mixture of 1 µM rotenone, 1 µg/mL antimycin A, 100 mM 2-deoxyglucose (100 mM) and 5 µg/ml Hoechst 33342. The OCR and ECAR were normalised to the cell count. The number of cells in each well was counted using the Cytation 3 Cell Imaging Reader (BioTek) and analysed using the Gen5 software (BioTek). Sample evaluations were performed in at least three independent experiments.

### NAD^+^/NADH

Cells (10 × 10^3^ per well) were seeded in 96-well plates, and the ratio of nicotinamide adenine dinucleotide (NAD^+^/NADH) was assessed using the NAD^+^/NADH Glo assay (Promega), following the manufacturer’s protocol for seeded cells.

### GSH detection assay

Cells (5 × 10^3^ per well) were seeded in 96-well plates, and GSH levels were assessed using the GSH/GSSG Glo assay (Promega) according to the manufacturer’s protocol for seeded cells.

### Data analysis, visualisation, and statistics

Figures were prepared, and statistics were calculated using GraphPad PRISM 10. Outliers were identified by the ROUT method and excluded from analysis. Data were presented as means with error bars representing the standard errors, unless otherwise indicated. One-sample unpaired t-test (2 groups), one-way ANOVA and two-way ANOVA tests (more than two groups) were utilised to calculate *p* values; a *p* value of less than 0.05 was considered significant. Statistical details can also be found in the figure legends. Blinding was not performed for the experimental groups. Synopsis, Figs. 1E,H and 3A and EV1D and EV2D; Appendix Fig. S1A were created with BioRender.com.

## Supplementary information

Table EV1

Table EV2

Table EV3

Appendix

Peer Review File

Source data Fig. 1

Source data Fig. 2

Source data Fig. 3

Source data Fig. 4

Source data Fig. 5

Expanded View Figures

## Data Availability

The FIJI macro used for image processing and analysis of lipid droplets is available at https://github.com/MracekLab-Prague/Lipid-droplet-analysis.git. The mass spectrometry proteomics data have been deposited in the ProteomeXchange Consortium via the PRIDE (Perez-Riverol et al, 2025) partner repository: https://www.ebi.ac.uk/pride/archive/projects/PXD069869. The source data of this paper are collected in the following database record: biostudies:S-SCDT-10_1038-S44319-026-00898-y.
