# Supplementary material for: Polyunsaturated fatty acid sequestration protects against mitochondrial dysfunction-induced ferroptosis
Source: EMBO Rep. 2026 Aug 14;27(18):5382–406. doi: 10.1038/s44319-026-00898-y (PMC13601559; doi:10.1038/s44319-026-00898-y)
Supplement: Supplementary file 1 [file 44319_2026_898_MOESM11_ESM.pdf]

Expanded View Figures

Figure EV1. Metabolic remodelling in CIV-deficient cells.

Metabolic profiling (LC–MS) of TCA cycle metabolites (**A**), 2-hydroxyglutarate (**B**), and serine (**C**) after 24 h incubation in fresh culture media. Data are expressed as fold changes compared to WT ($n = 3$). (**D**) Schematic representation of de novo lipid synthesis. (**E**) Glycerol 3-phosphate (G3P) labelling from $^{13}C_6$-glucose after 24 h incubation ($n = 3$). (**F**) ^{13}C labelling of palmitic acid after 24 h incubation with $^{13}C_6$-glucose or $^{13}C_5$-glutamine measured by LC–MS after fatty acyl chain lipolysis ($n = 3$). (**G**) Metabolic modelling of the flux of de novo synthesised palmitic acid into TG (1-palmitoyl-2-oleoyl-sn-glycero-3-phosphocholine; TG 16:0_18:1_18:1) or phosphatidylcholine (PC 16:0_18:1). Data were expressed as fold changes relative to control ($n = 6$). *N* represents the number of biological replicates. Bar graphs represent means ± SD. One-way (**B**, **C**) or two-way (**A**, **F**) ANOVA was performed (**A**–**C** – $p^{****} < 0.0001$; **E** – $p^{*} = 0.0124$, $p^{**} = 0.0017$; **F** – $p^{*} = 0.0024$; $p^{****} < 0.0001$).

© The Author(s)

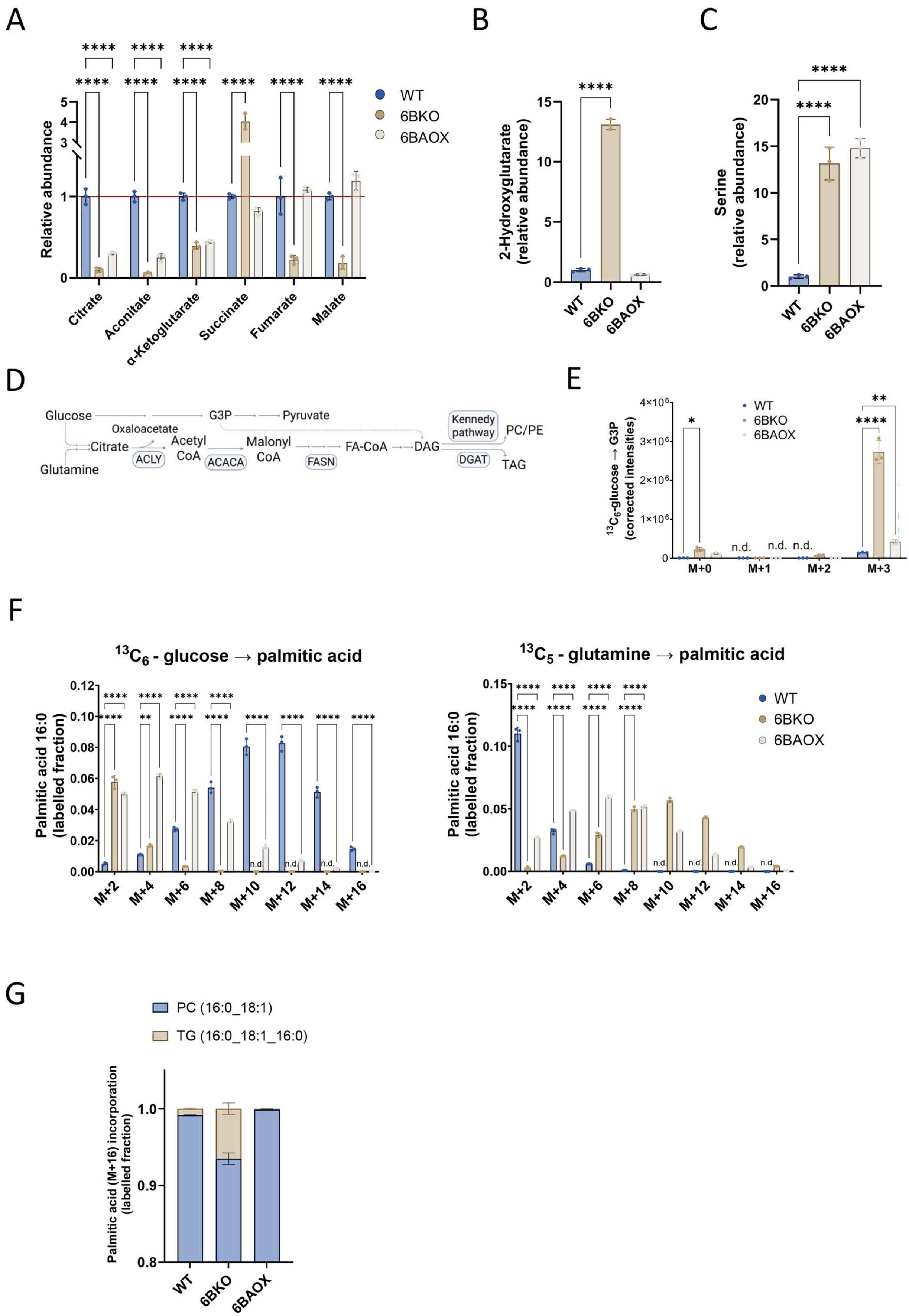
A
B
C
D
E
F
G
Relative abundance
Citrate
Aconitate
α-Ketoglutarate
Succinate
Fumarate
Malate
WT
6BKO
6BAOX
2-Hydroxyglutarate (relative abundance)
Serine (relative abundance)
Glucose
G3P
Pyruvate
Oxaloacetate
Citrate
Glutamine
ACLY
Acetyl CoA
ACACA
Malonyl CoA
FASN
FA-CoA
DAG
Kennedy pathway
PC/PE
DGAT
TAG
13C6-glucose → G3P (corrected intensities)
M+0
M+1
M+2
M+3
n.d.
13C6 - glucose → palmitic acid
13C5 - glutamine → palmitic acid
Palmitic acid 16:0 (labelled fraction)
PC (16:0_18:1)
TG (16:0_18:1_16:0)
Palmitic acid (M+16) incorporation (labelled fraction)

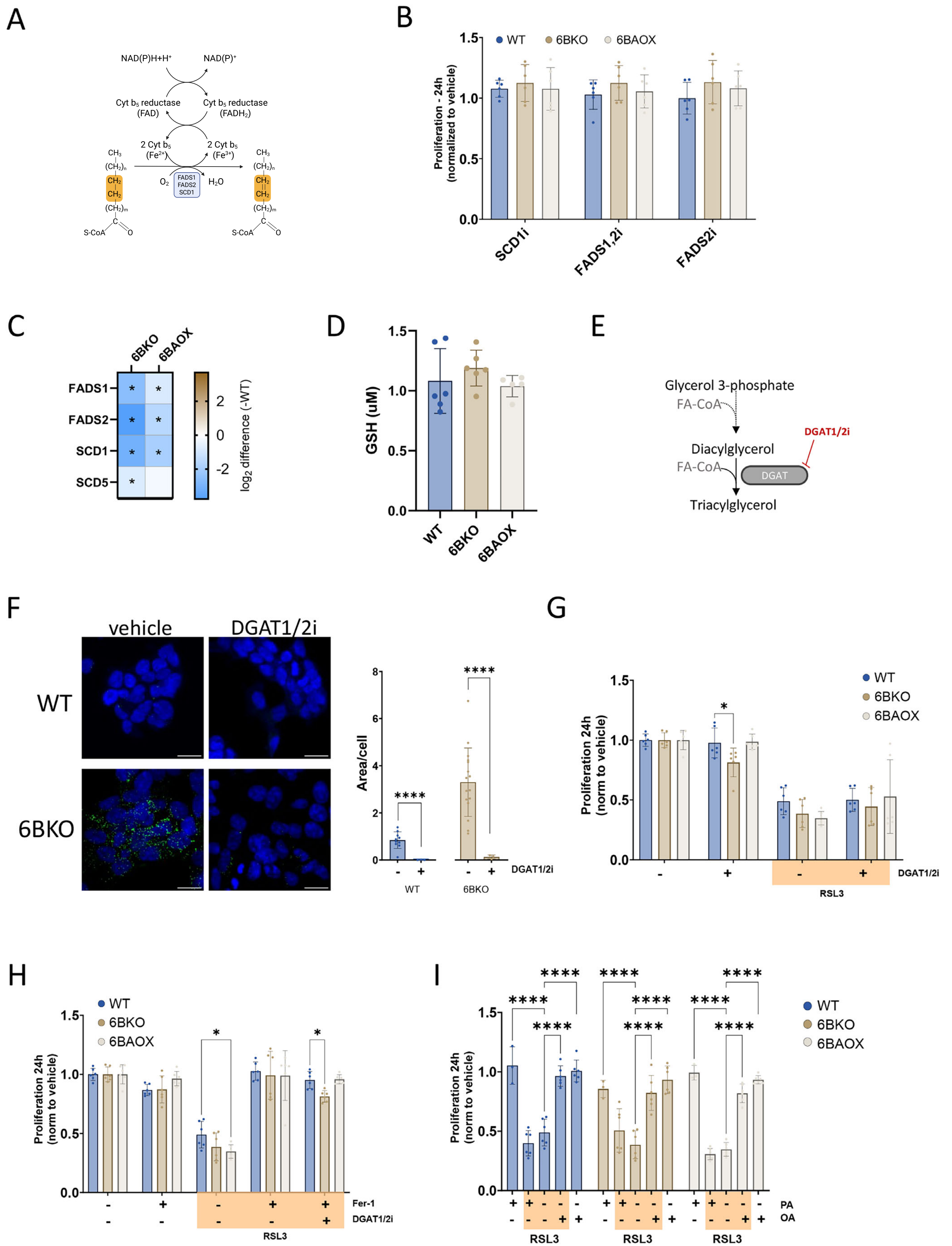
A
NAD(P)H+H+
NAD(P)+
Cyt b5 reductase (FAD)
Cyt b5 reductase (FADH2)
2 Cyt b5 (Fe2+)
2 Cyt b5 (Fe3+)
O2
FADS1 FADS2 SCD1
H2O
S-CoA
B
WT 6BKO 6BAOX
Proliferation - 24h (normalized to vehicle)
SCD1i
FADS1,2i
FADS2i
C
6BKO 6BAOX
FADS1
FADS2
SCD1
SCD5
log2 difference (-WT)
D
GSH (uM)
WT 6BKO 6BAOX
E
Glycerol 3-phosphate
FA-CoA
Diacylglycerol
DGAT1/2i
DGAT
Triacylglycerol
F
vehicle
DGAT1/2i
WT
6BKO
Area/cell
DGAT1/2i
G
Proliferation 24h (norm to vehicle)
RSL3
H
Fer-1
DGAT1/2i
RSL3
I
PA
OA
RSL3

Figure EV2. PUFA stress response in CIV-deficient cells.

(**A**) Scheme of fatty acyl chain desaturation. (**B**) Cell proliferation analysis of the cell lines incubated for 24 h with inhibitors against SCD1 (CAY10566, 500 nM), FADS1 and 2 (CP-24879, 30 μM) and FADS2 (SC-26196, 10 μM). Data were expressed as fold change to vehicle ($n = 6$). (**C**) LFQ-MS analysis of the cellular desaturase levels. The heatmap displays $\log_2$(fold change); asterisks indicate proteins that are significantly altered (p value <0.05, $n = 3$). (**D**) GSH level assessed in WT, 6BKO and 6BAOX ($n = 6$). Data were expressed as fold change relative to WT. (**E**) Schematic representation of TG formation. (**F**) Representative image and quantification of BODIPY 493/503 staining in WT and 6BKO cell line treated with vehicle or a mixture of DGAT1 (A922500, 1 μM) and DGAT2 (PF-06424439, 10 μM) inhibitors (DGAT1/2i) ($n \geq 3$). Scale bar size is 20 μm. (**G–I**) Proliferation analysis of the cell lines incubated for 24 h with RSL3 (1 μM). The cells were pretreated for 48 h with DGAT1/2i (**G**, **H**), or for 24 h with the ferroptosis inhibitor ferrostatin-1 (Fer-1, 200 nM) (**H**), BSA-conjugated oleic (OA, 25 μM) or palmitic (PA, 25 μM) acid (**I**). Data were expressed as fold change to vehicle ($n = 6$). *N* represents the number of biological replicates. Bar graphs represent means ± SD. Two-way (**B**, **G–I**), one-way (**D**) ANOVA, or *t*-test (**F**) was performed (**F**– p^{****}0.0001; **G** – $p^{*} = 0.0350$; **H** – $p^{*} = 0.0203$ (RSL3), 0.0240 (RSL3 + DGAT1/2i+Fer-1); **I** – $p^{****} < 0.0001$).

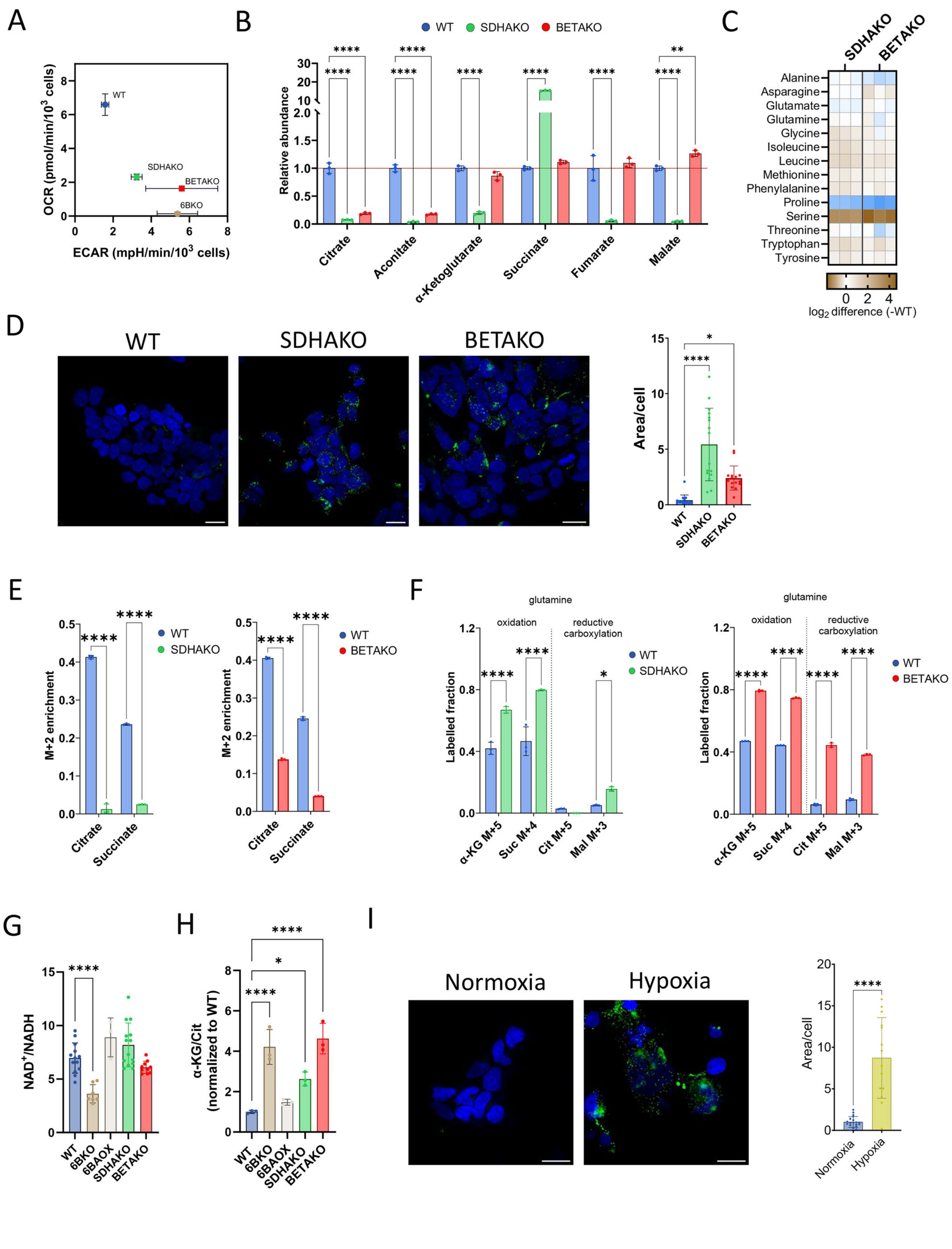
A
OCR (pmol/min/10^3 cells)
ECAR (mpH/min/10^3 cells)
WT
SDHAKO
BETAKO
6BKO
B
WT
SDHAKO
BETAKO
Relative abundance
****
**
Citrate
Aconitate
α-Ketoglutarate
Succinate
Fumarate
Malate
C
SDHAKO
BETAKO
Alanine
Asparagine
Glutamate
Glutamine
Glycine
Isoleucine
Leucine
Methionine
Phenylalanine
Proline
Serine
Threonine
Tryptophan
Tyrosine
log$_2$ difference (-WT)
D
WT
SDHAKO
BETAKO
Area/cell
****
*
E
M+2 enrichment
WT
SDHAKO
BETAKO
Citrate
Succinate
F
glutamine
oxidation
reductive carboxylation
Labelled fraction
WT
SDHAKO
BETAKO
α-KG M+5
Suc M+4
Cit M+5
Mal M+3
G
NAD$^+$/NADH
WT
6BKO
6BAOX
SDHAKO
BETAKO
H
α-KG/Cit (normalized to WT)
WT
6BKO
6BAOX
SDHAKO
BETAKO
I
Normoxia
Hypoxia
Area/cell
****

 © The Author(s)

Figure EV3. Metabolic remodelling in OXPHOS-limited cells.

(**A**) Metabolic phenotype of WT, 6BKO, BETAKO (data published in (Cunatova et al, 2024)) and SDHAKO cells represented as a phenogram – combined parallel evaluation of cellular oxygen consumption rate (OCR) and extracellular acidification rate (ECAR). Data were expressed as means ± SEM ($n \geq 3$). (**B**) Metabolic profiling (LC–MS) of TCA cycle metabolites after 24 h incubation in fresh culture media. Data are expressed as fold changes compared to WT ($n = 3$). (**C**) Heatmap showing differences in amino acid levels in SDHAKO and BETAKO cells after 24 h of incubation in culture media. Data represent $\log_2$(fold change) compared to WT ($n = 3$). (**D**) Representative image and quantification of BODIPY 493/503 staining of the WT, SDHAKO and BETAKO cell lines ($n \geq 15$). Scale bar size is 20 µm. (**E**, **F**) The WT, SDHAKO and BETAKO cells were incubated with the tracer ($^{13}C_6$-glucose or $^{13}C_5$-glutamine) for 24 h, and the labelled fraction (proportion of total pool) of TCA cycle metabolites derived from $^{13}C_6$-glucose (**E**) or $^{13}C_5$-glutamine (**F**) was assessed by LC–MS ($n = 3$). (**G**) NAD^+/NADH ratio assessed in WT, 6BKO, 6BAOX, BETAKO (data published in (Cunatova et al, 2024)) and SDHAKO ($n \geq 3$). (**H**) α-ketoglutarate/citrate (α-KG/Cit) ratio measured by LC–MS after 24 h incubation in fresh culture media. Data were expressed as fold changes compared to WT ($n = 3$). (**I**) Representative image and quantification of BODIPY 493/503 staining of the WT in normoxia or hypoxia ($n \geq 15$). Scale bar size is 20 µm. *N* represents the number of biological replicates. Bar graphs represent means ± SD. Two-way (**B**, **E**, **F**), one-way (**D**, **G**) ANOVA or *t*-test (**I**) was performed (**B** – $p^{**} = 0.0015$; **D** – $p^{*} = 0.0162$; **F** – $p^{*} = 0.0115$; **H** – $p^{*} = 0.0139$; $p^{****} < 0.0001$).
