# Supplementary material for: Polyunsaturated fatty acid sequestration protects against mitochondrial dysfunction-induced ferroptosis
Source: EMBO Rep. 2026 Aug 14;27(18):5382–406. doi: 10.1038/s44319-026-00898-y (PMC13601559; doi:10.1038/s44319-026-00898-y)
Supplement: Supplementary file 2 [file 44319_2026_898_MOESM4_ESM.pdf]

Appendix

Polyunsaturated fatty acid sequestration protects against ferroptosis in mitochondrial dysfunction

Table of contents:

Appendix Figure S1: Metabolic remodelling in CIV-deficient cells 2

Appendix Figure S2: Relative levels of PC, PE and TG containing at least one PUFA in OXPHOS-deficient cells 3

Appendix Figure S3: PUFA stress response in the COX6B1 addback cell line 4-5

Appendix Figure S4: PUFA-mediated stress response in CIV-deficient cells 5

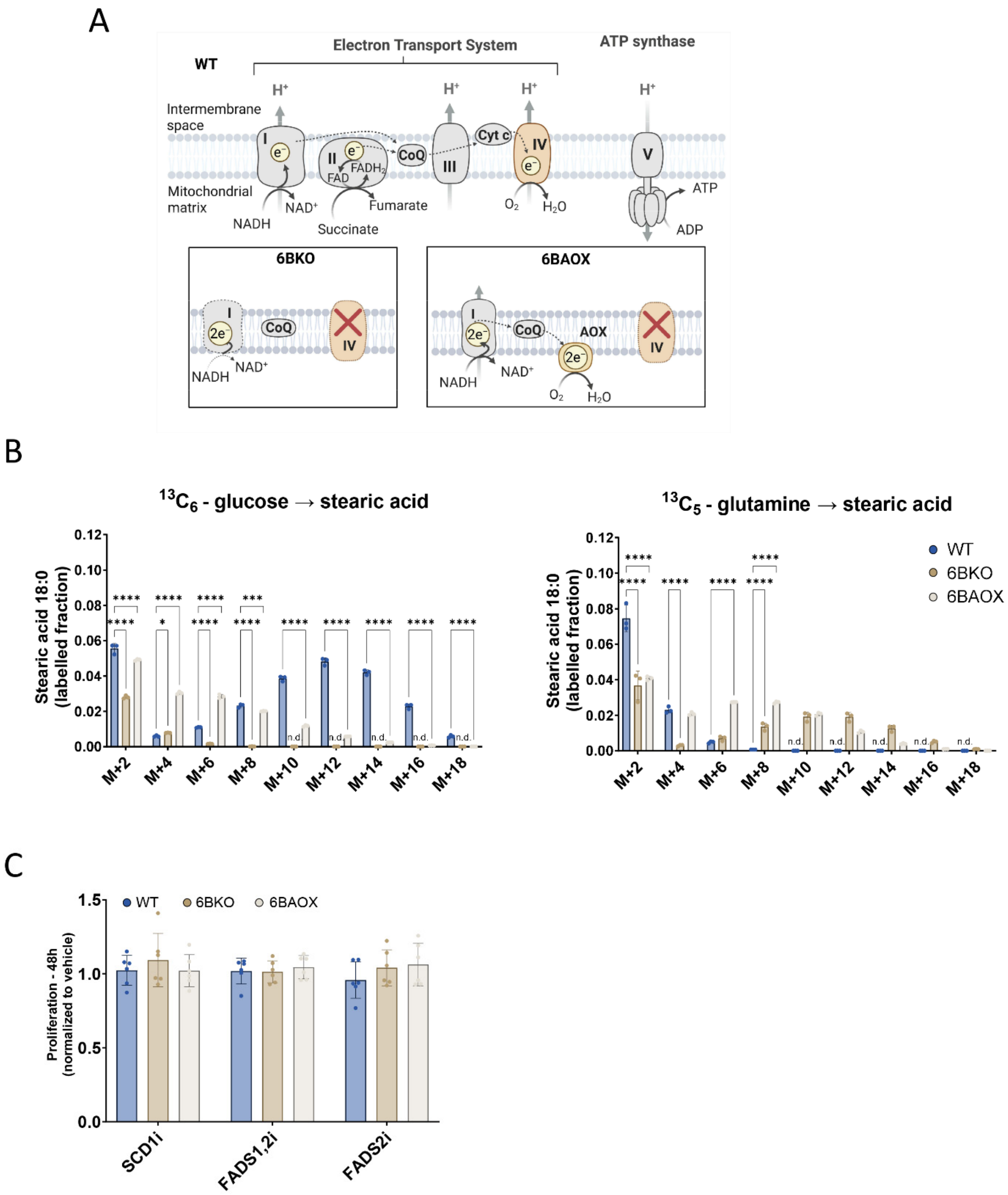

Appendix Figure S1: Metabolic remodelling in CIV-deficient cells

(A) Scheme of the OXPHOS system in the models with knock-out of the COX6B1 subunit and the knock-out model overexpressing alternative oxidase (AOX). (B) ^{13}C labelling of stearic acid after 24h incubation with $^{13}C_6$-glucose or $^{13}C_5$-glutamine measured by lipidomic analysis after fatty acyl chain lipolysis (n = 3). (C) Cell proliferation analysis of the cell lines incubated for 48h with inhibitors against SCD1 (SCD1i), FADS1 and 2 (FADS1,2i). Data are expressed as fold change to vehicle (n = 6). N represents the number of biological replicates. Bar graphs represent means ± SD. 2-way ANOVA was performed (B – p*=0.0246, p***=0.0003; p**** <0.0001).

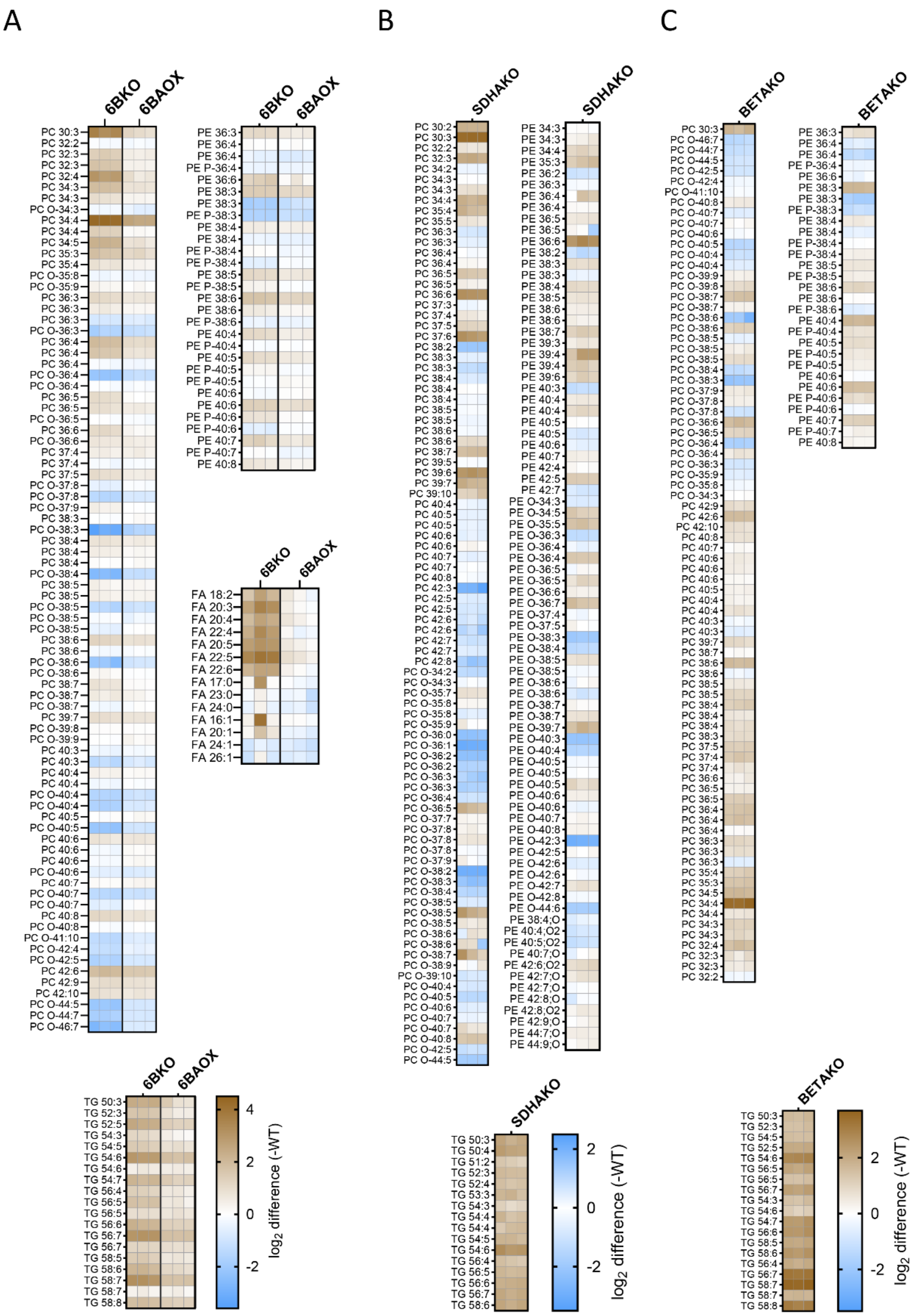

Appendix Figure S2: Relative levels of PC, PE and TG containing at least one PUFA in OXPHOS-deficient cells

The heatmaps show relative levels of PC, PE and TG containing at least one PUFA in 6BKO and 6BAOX (A), SDHAKO (B), and BETAKO (C) cells. Relative levels of free fatty acids (FA) in 6BKO and 6BAOX (PUFA contains ≥ 2 double bonds) are shown in (A). Data are expressed as log_2(fold change) compared to WT (n = 3, biological replicates).

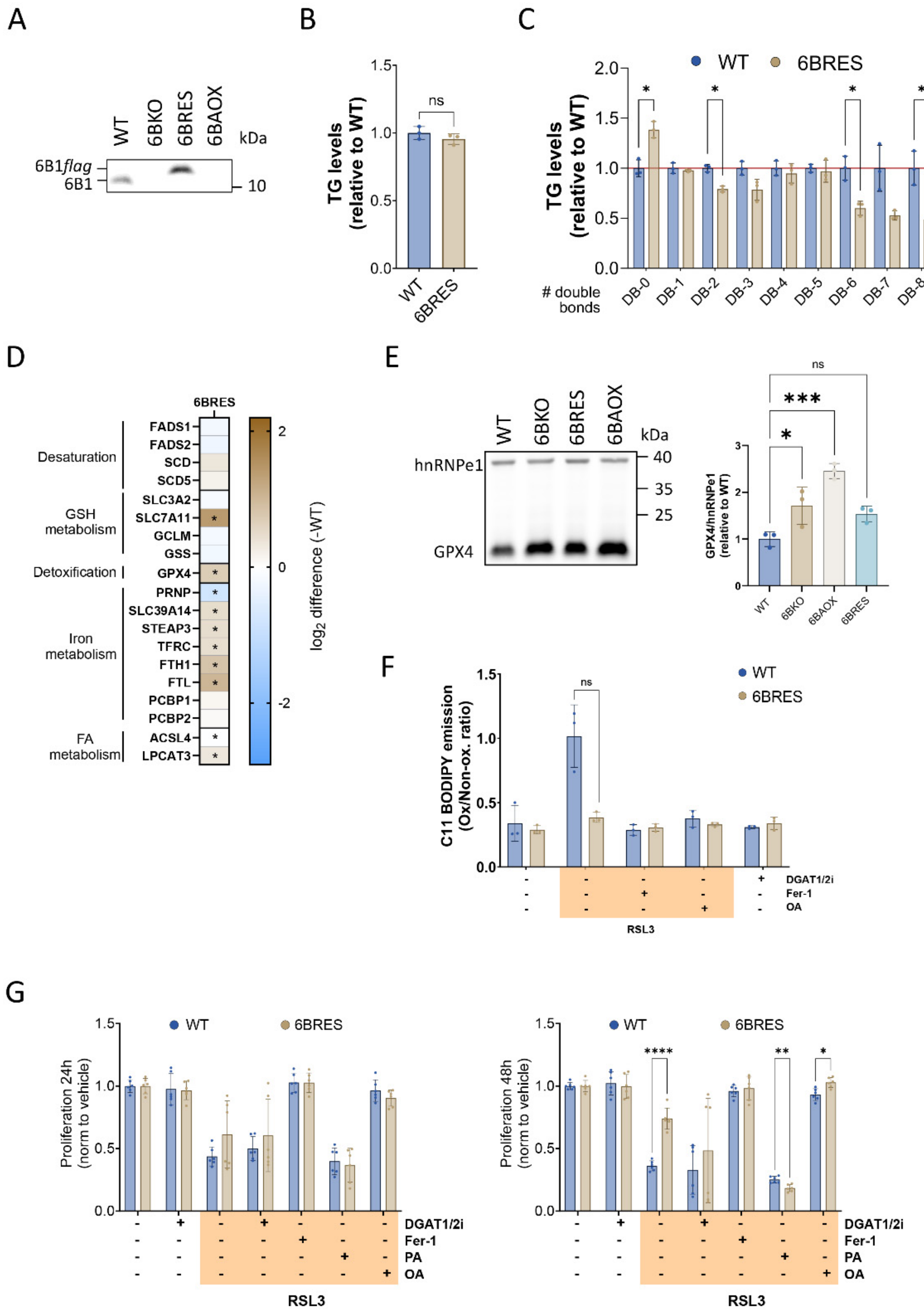

Appendix Figure S3: PUFA stress response in the COX6B1 addback cell line

(A) Representative SDS-PAGE–WB analysis of the protein steady-state level of the COX6B1 subunit in whole-cell lysates of WT, 6BKO, 6BRES and 6BAOX. FLAG-tagged COX6B1 (6B1*flag*) and endogenous COX6B1 (6B1) proteins are marked. Relative level of TG (B) and relative levels of TGs differentiated by double-bond content (C) measured by LC–MS (labelled as in Figure 2B). Data are expressed as fold change relative to WT (n = 3). (D) LFQ-MS analysis of ferroptosis-related protein levels. The heatmap displays log_2(fold change) values for 6BRES relative to WT (n = 3); asterisks denote proteins that have changed significantly (p-value < 0.05). (E) Representative SDS-PAGE/WB and quantification of the protein steady-state level of GPX4 using hnRNPe1 as the loading control in whole-cell lysates of WT, 6BKO, 6BRES and 6BAOX. The quantified GPX4 signal was normalised to hnRNPe1, and the data are

expressed relative to WT (n = 3). (F) Lipid peroxidation assessed by lipid peroxidation sensor BODIPY 581/591 C11 (n = 3) in WT and 6BRES cell lines incubated for 24h with a ferroptosis inducer RSL3 (1µM). The cells were pretreated for 48 hours with a mixture of DGAT1 (A922500, 1 µM) and DGAT2 (PF-06424439, 10 µM) inhibitors (DGAT1/2i), or for 24h with the ferroptosis inhibitor ferrostatin-1 (Fer-1, 200 nM). (F-G) Proliferation analysis of the cell lines incubated for 24h (F) or 48h (G) with RSL3 (1µM). The cells were pretreated for 48 hours with DGAT1/2i, for 24 hours with the ferroptosis inhibitor Fer-1, or BSA-conjugated OA or PA (25 µM). Data are expressed as fold change to vehicle (n = 3).

N represents the number of biological replicates. Bar graphs represent means ± SD. T-test (B, C, F, G) or one-way (E) was performed (C – p*=0.0368 (DB-0), 0.0133 (DB-2), 0.0483 (DB-6, 8), 0.048; E – p*=0.0186, p***=0.0002; G – p*=0.0165, p**=0.0089; p**** <0.0001).

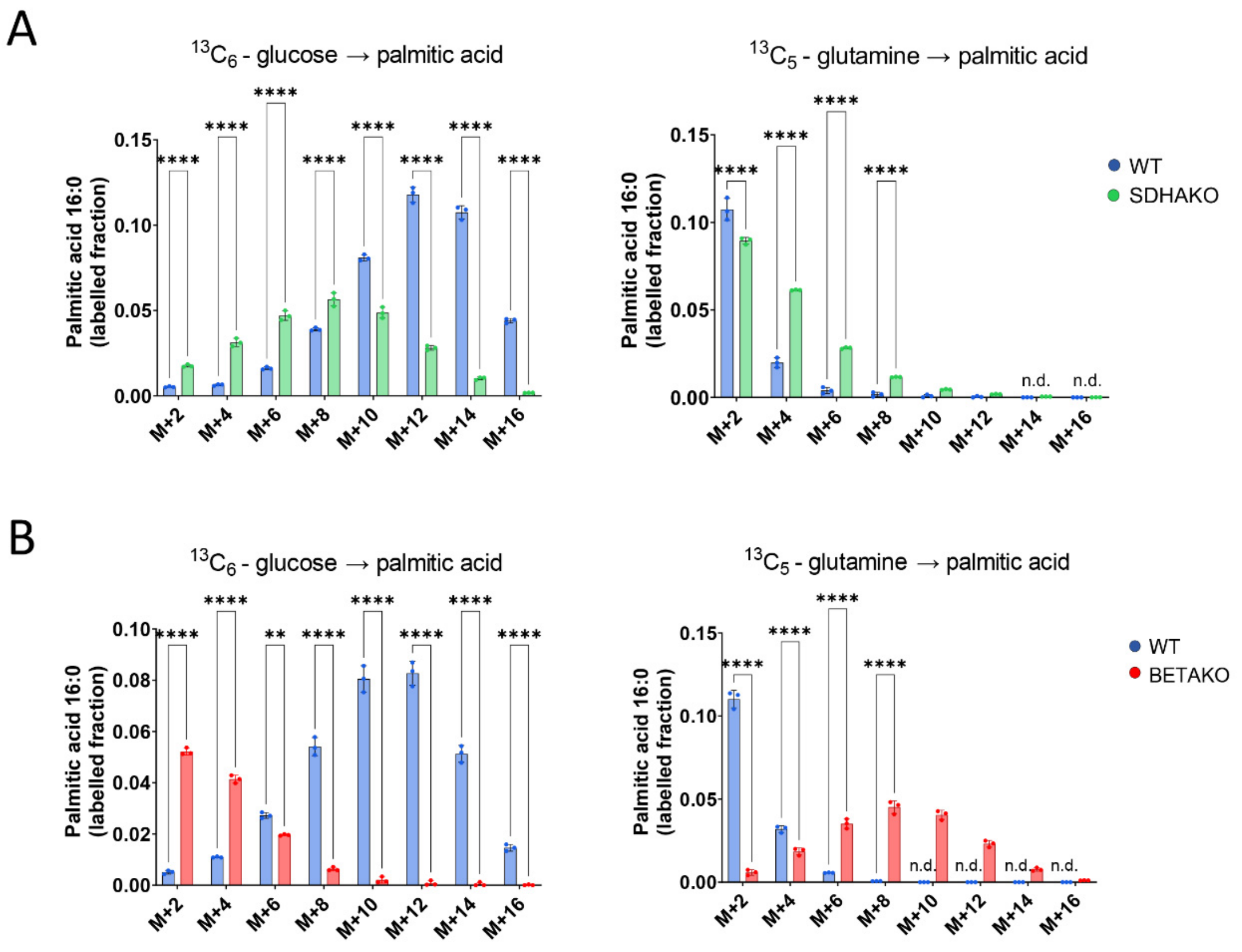

Appendix Figure S4: PUFA-mediated stress response in CIV-deficient cells

^{13}C labelling of palmitic acid in SDHAKO (A) or BETAKO (B) cells (n = 3). The WT, SDHAKO and BETAKO cells were incubated with the tracer ($^{13}C_6$-glucose or $^{13}C_5$-glutamine) for 24h, and the labelled fraction of palmitic acid was measured after fatty acyl chain lipolysis by LC-MS.

N represents the number of biological replicates. Bar graphs represent means ± SD. 2-way ANOVA was performed (B – p**=0.0021; p**** <0.0001).
