# Supplementary material for: Polyunsaturated fatty acid sequestration protects against mitochondrial dysfunction-induced ferroptosis
Source: EMBO Rep. 2026 Aug 14;27(18):5382–406. doi: 10.1038/s44319-026-00898-y (PMC13601559; doi:10.1038/s44319-026-00898-y)
Supplement: Supplementary file 3 [file 44319_2026_898_MOESM5_ESM.pdf]

EMBO reports

Polyunsaturated fatty acid sequestration protects against mitochondrial dysfunction-induced ferroptosis

Alena Pecinova, Guillermo Puertas-Frias, María José Saucedo-Rodríguez, Kristýna Čunátová, Lukáš Alán, Patrik Hrbáč, Michal Kněžů, Soňa Koníčková, Jakub Schimmer, Monika Krakovková, Marek Vrbacký, Tomas Cajka, Ondrej Kuda, Erika Fernández-Vizarra, Massimo Zeviani, Hana Hansíková, Tomáš Honzík, Josef Houštěk, Petr Pecina, and Tomas Mráček

Corresponding author(s): Alena Pecinova (alena.pecinova@fgu.cas.cz)

Review Timeline:

Submission Date:	12th Nov 25
Editorial Decision:	26th Jan 26
Revision Received:	12th May 26
Editorial Decision:	19th Jun 26
Revision Received:	16th Jul 26
Accepted:	29th Jul 26

Editor: Martina Rembold

Transaction Report:

(Note: With the exception of the correction of typographical or spelling errors that could be a source of ambiguity, letters and reports are not edited. Depending on transfer agreements, referee reports obtained elsewhere may or may not be included in this compilation. Referee reports are anonymous unless the Referee chooses to sign their reports.)

1st Editorial Decision 26th Jan 2026

Dear Dr. Pecinova

Thank you for the submission of your manuscript to EMBO reports. I apologize for the delay in handling it, but we have now received the full set of referee reports as well as referee cross-comments that are all pasted below.

As you will see, referee #1 and #3 acknowledge that the findings are potentially interesting and consider the data overall solid but they also raise a number of concerns and have suggestions how to strengthen your study, which need to be addressed.

We realize that it is difficult to revise to a specific deadline. In the interest of protecting the conceptual advance provided by the work, we recommend a revision within 3 months (28th Apr 2026). Please discuss the revision progress ahead of this time with the editor if you require more time to complete the revisions.

You can either publish the study as a short report or as a full article. For short reports, the revised manuscript should not exceed 27,000 characters (including spaces but excluding materials & methods and references) and 5 main plus 5 expanded view figures. The results and discussion sections must further be combined, which will help to shorten the manuscript text by eliminating some redundancy that is inevitable when discussing the same experiments twice. For a normal article there are no length limitations, but it should have more than 5 main figures and the results and discussion sections must be separate. In both cases, the entire materials and methods must be included in the main manuscript file.

IMPORTANT NOTE: we perform an initial quality control of all revised manuscripts before re-review. Your manuscript will FAIL this control and the handling will be DELAYED if the following APPLIES:
1) A data availability section providing access to data deposited in public databases is missing. If you have not deposited any data, please add a sentence to the data availability section that explains that.
2) Your manuscript contains statistics and error bars based on n=2. Please use scatter blots in these cases. No statistics should be calculated if n=2.

When submitting your revised manuscript, please review the submission guidelines (https://link.springer.com/journal/44319/submission-guidelines#cms-Revised-submissions) and carefully review the instructions that follow below. Failure to include requested items will delay the evaluation of your revision.

1) a .docx formatted version of the manuscript text (including legends for main figures, EV figures and tables). Please make sure that the changes are highlighted to be clearly visible.

2) individual production quality figure files as .eps, .tif, .jpg (one file per figure). See hhttps://media.springernature.com/original/springer-cms/rest/v1/content/27825798/data/v1 for more info on how to prepare your figures.

3) We replaced Supplementary Information with Expanded View (EV) Figures and Tables that are collapsible/expandable online. A maximum of 5 EV Figures can be typeset. EV Figures should be cited as 'Figure EV1, Figure EV2" etc... in the text and their respective legends should be included in the main text after the legends of regular figures.

- For the figures that you do NOT wish to display as Expanded View figures, they should be bundled together with their legends in a single PDF file called *Appendix*, which should start with a short Table of Content. Appendix figures should be referred to in the main text as: "Appendix Figure S1, Appendix Figure S2" etc. See 'Expanded View' section of the submission guidelines.

- Additional Tables/Datasets should be labeled and referred to as Table EV1, Dataset EV1, etc. Legends have to be provided in a separate tab in case of .xls files. Alternatively, the legend can be supplied as a separate text file (README) and zipped together with the Table/Dataset file.

4) a .docx formatted letter INCLUDING the reviewers' reports and your detailed point-by-point responses to their comments. As part of the EMBO Press transparent editorial process, the point-by-point response is part of the Review Process File (RPF), which will be published alongside your paper.

5) a complete author checklist, which you can download from our author guidelines. Please insert information in the checklist that is also reflected in the manuscript. The completed author checklist will also be part of the RPF.

6) Please note that all corresponding authors are required to supply an ORCID ID for their name upon submission of a revised manuscript (<https://orcid.org/>), which can be linked to your profile in the manuscript submission system.

7) Before submitting your revision, primary datasets produced in this study need to be deposited in an appropriate public database. Please remember to provide a reviewer password if the datasets are not yet public. The accession numbers and database should be listed in a formal "Data Availability" section placed after Methods. Please note that the Data Availability

Section is restricted to new primary data that are part of this study. * Note - All links should resolve to a page where the data can be accessed. *

If your study has not produced novel datasets, please mention this fact in the Data Availability Section.

8) At EMBO Press we ask authors to provide source data for the main manuscript figures. You will receive a separate email with instructions for providing source data with your revised manuscript, including how to upload and organize the files.

9) Our journal also encourages inclusion of *data citations in the reference list* to directly cite datasets that were re-used and obtained from public databases. Data citations in the article text are distinct from normal bibliographical citations and should directly link to the database records from which the data can be accessed. In the main text, data citations are formatted as follows: "Data ref: Smith et al, 2001" or "Data ref: NCBI Sequence Read Archive PRJNA342805, 2017". In the Reference list, data citations must be labeled with "[DATASET]". A data reference must provide the database name, accession number/identifiers and a resolvable link to the landing page from which the data can be accessed at the end of the reference.

10) Regarding data quantification, the following points must be specified in each figure legend:

- the name of the statistical test used to generate error bars and P values,

- the number (n) of independent experiments (please specify technical or biological replicates) underlying each data point,

- the nature of the bars and error bars (s.d., s.e.m.),

- If the data are obtained from n Program fragment delivered error ``Can't locate object method "less" via package "than" (perhaps you forgot to load "than"?) at //ejpvfs23/sites23b/embor_www/letters/embor_decision_revise_and_review.txt line 54.' 2, use scatter blots showing the individual data points.

Discussion of statistical methodology can be reported in the materials and methods section, but figure legends should contain a basic description of n, P and the test applied.

- Please also include scale bars in all microscopy images.

11) The journal requires a statement specifying whether or not authors have competing interests (defined as all potential or actual interests that could be perceived to influence the presentation or interpretation of an article). In case of competing interests, this must be specified in your disclosure statement.

12) All Materials and Methods need to be described in the main text using our 'Structured Methods' format, which is required for all research articles. According to this format, the Methods section includes a Reagents and Tools Table (listing key reagents, experimental models, software and relevant equipment and including their sources and relevant identifiers) followed by a Methods and Protocols section describing the methods using a step-by-step protocol format. The aim is to facilitate adoption of the methodologies across labs. More information on how to adhere to this format as well as a downloadable template (.docx) for the Reagents and Tools Table can be found in our author guidelines.

We would also welcome the submission of cover suggestions, or motifs to be used by our Graphics Illustrator in designing a cover.

As part of the EMBO publication's Transparent Editorial Process, EMBO reports publishes online a Review Process File (RPF) to accompany accepted manuscripts. This File will be published in conjunction with your paper and will include the referee reports, your point-by-point response and all pertinent correspondence relating to the manuscript.

You are able to opt out of this by letting the editorial office know (emboreports@embo.org). If you do opt out, the Review Process File link will point to the following statement: "No Review Process File is available with this article, as the authors have chosen not to make the review process public in this case."

I look forward to seeing a revised form of your manuscript when it is ready.

Yours sincerely,

Martina Rembold, PhD
Senior Editor
EMBO reports

==================

Referee #1:

Comments on "Polyunsaturated fatty acid sequestration protects against ferroptosis in mitochondrial dysfunction" by Puertas-Frias at al.
The authors demonstrate that the polyunsaturated fatty acid (PUFA) stress response is a key adaptive mechanism in cells with impaired oxidative phosphorylation (OXPHOS). In this context, cells mitigate PUFA-induced toxicity by redirecting PUFAs into triacylglycerols (TGs), which are subsequently stored in lipid droplets (LDs). Additional consequences include suppression of desaturase expression and upregulation of GPX4. These findings are supported by genetic models of deficiencies in complex II, IV, and V, by hypoxic conditions, and by primary fibroblasts from mitochondrial disease patients, as well as by the plasma of patients with MERRF syndrome. Overall, the data presented are relevant to the metabolic field and support the literature linking mitochondrial deficiency to lipid remodeling as a mechanism of ferroptosis regulation. The study is well suited for publication in EMBO Reports pending clarification of some points.
Major concerns:
1. Based on glucose labeling (Fig. 2B, C) and glutamine labeling (Fig. 2E), overexpression of AOX in COX6B1 KO cells did not fully restore the functionality of the TCA cycle and OXPHOS. Additionally, the primary source of de novo lipid synthesis remains altered in AOX cells (Fig. 3, and TG levels in Figs. 4 and 5). Some metabolic markers associated with ferroptosis are also altered in AOX and COX6B1 KO cells (Fig. 5), which could represent a misinterpretation of some findings. Therefore, an additional proof-of-concept experiment using COX6B1 KO cells with COX6B1 addback would provide a more physiological comparison.
2. Are the effects of RSL3 on cell proliferation (Fig. 5) due to the already known lower proliferation capacity of COX6B1 KO cells? Using a cell death measurement instead of proliferation or metabolic activity would be more relevant to the conclusion. Also, including the relevant controls for lipid peroxidation evaluation with and without cotreatment with radical-trapping agents would be helpful.
3. Many of the conclusions are based on MS data without further validation. Therefore, it is relevant to evaluate mitochondrial activity in live cells by measuring oxygen and ATP levels. Additionally, quantifying mitochondrial number per cell, measuring cytochrome c oxidase activity, and determining the NAD+/NADH ratio in the cellular models are relevant to strengthen the manuscript.

Minor points:
4. As observed in Fig. 1B, COX6B1 KO cells have increased doubling time compared to WT or AOX OE. Consider proliferation rate to perform flux analysis?
5. Accumulation of 2-hydroxyglutarate: measurement?
6. TG accumulation: Are free fatty acids accumulated in both COX6B1 KO and AOX?
7. Lipid droplets measurement: explain the normalization of Fig. 3C, 6C, 7B, 8C
8. Relevant Mass spec data validation by immunoblotting.
9. Statistics analysis in graphs of Fig. 7
10. Improve read flow of the discussion section, focusing on the strength, novelty and implications of the presented data.

Referee #2:

The presented manuscript is a technical description of metabolic reprogramming in a number of enzyme knockouts. The data presented in the paper is interesting and generally rigorously collected and presented but does not really address the title, abstract, or fundamental claims of the paper. The title, abstract, and highlights ALL present claims about PUFA metabolism, but the only data examining PUFA metabolism is lipidomics analysis of complex lipids including TGs. No causal experiments are conducted that support the actual claims of the paper.

Referee #3:

The manuscript examines how cells with impaired mitochondrial oxidative phosphorylation (OXPHOS) adapt lipid metabolism to preserve membrane integrity and avoid ferroptosis. Using genetic models of mitochondrial complex II, IV, and V deficiencies, hypoxia, patient-derived fibroblasts, and plasma samples from individuals with MERRF syndrome, the authors identify a conserved "PUFA stress response" characterized by sequestration of polyunsaturated fatty acids (PUFAs) into triacylglycerols stored in lipid droplets, downregulation of fatty acid desaturases, and upregulation of GPX4. The study integrates metabolomics, lipidomics, isotope tracing, proteomics, and functional assays to propose PUFA sequestration as a protective adaptation in OXPHOS-compromised cells.
The authors have done a thorough job citing the existing literature.
Major Comments
The work provides a coherent and mechanistically plausible framework linking mitochondrial dysfunction to lipid remodeling as a defense against ferroptosis. Demonstrating that PUFA sequestration into triacylglycerols is conserved across multiple OXPHOS

defects, hypoxia, and patient-derived fibroblast and blood, represents a meaningful conceptual advance with relevance to mitochondrial biology and regulated cell death.
A major strength is the complementary approaches and the use of multiple genetic OXPHOS-deficient models. Normalization of phenotypes by AOX expression in the CIV-deficient cells strengthens the causal link between impaired electron transport and lipid remodeling. The distinction between acute versus sustained OXPHOS dysfunction and its impact on fatty acid desaturation is well reasoned.
The functional assays using GPX4 inhibition, DGAT inhibition, ferrostatin-1 rescue, and fatty acid supplementation support the conclusion that PUFA sequestration protects against ferroptosis. While proliferation-based readouts are informative, inclusion of direct lipid peroxidation measurements would further strengthen the mechanistic link, particularly in the DGAT inhibition experiments.
The inclusion of patient-derived fibroblasts and plasma lipidomics from MERRF patients is an important strength. The elevated PUFA-enriched circulating triacylglycerols is intriguing, but given cohort size and heterogeneity, these findings should be interpreted as hypothesis-generating and require validation in larger studies.
Minor Comments
Additional LC-MS methodological details and quality control metrics would improve reproducibility. Clarification of lipid droplet quantification normalization would be helpful. A brief definition of the term "PUFA stress response" early in the Results would help.

Response to the reviewers' comments regarding the manuscript EMBOR-2025-63174-T: "Polyunsaturated fatty acid sequestration protects against ferroptosis in mitochondrial dysfunction"

To meet the requirements of EMBO Reports and reviewers, the main changes include rewriting the Results and Discussion and consolidating figures into five main figures, three expanded-view figures, and four figures in the appendix PDF. Furthermore, the Materials and Methods have been expanded with additional methods, experimental details and quality control metrics to improve reproducibility.

Referee #1:

Comments on "Polyunsaturated fatty acid sequestration protects against ferroptosis in mitochondrial dysfunction" by Puertas-Frias at al. The authors demonstrate that the polyunsaturated fatty acid (PUFA) stress response is a key adaptive mechanism in cells with impaired oxidative phosphorylation (OXPHOS). In this context, cells mitigate PUFA-induced toxicity by redirecting PUFAs into triacylglycerols (TGs), which are subsequently stored in lipid droplets (LDs). Additional consequences include suppression of desaturase expression and upregulation of GPX4. These findings are supported by genetic models of deficiencies in complex II, IV, and V, by hypoxic conditions, and by primary fibroblasts from mitochondrial disease patients, as well as by the plasma of patients with MERRF syndrome. Overall, the data presented are relevant to the metabolic field and support the literature linking mitochondrial deficiency to lipid remodeling as a mechanism of ferroptosis regulation. The study is well suited for publication in EMBO Reports pending clarification of some points.

Major concerns:

1. Based on glucose labeling (Fig. 2B, C) and glutamine labeling (Fig. 2E), overexpression of AOX in COX6B1 KO cells did not fully restore the functionality of the TCA cycle and OXPHOS. Additionally, the primary source of de novo lipid synthesis remains altered in AOX cells (Fig. 3, and TG levels in Figs. 4 and 5). Some metabolic markers associated with ferroptosis are also altered in AOX and COX6B1 KO cells (Fig. 5), which could represent a misinterpretation of some findings. Therefore, an additional proof-of-concept experiment using COX6B1 KO cells with COX6B1 addback would provide a more physiological comparison.

We agree with the reviewer that AOX overexpression did not fully restore TCA cycle and OXPHOS function. However, full complementation was not the intention of the AOX model. The 6BAOX cell line was used as a model to attenuate mitochondrial redox stress, while retaining partial OXPHOS deficiency (due to lower H^+/e^- efficiency caused by loss of coupling sites at complexes III and IV), but this rationale was not adequately explained in the original manuscript. To address this, we added the following text into the Results "CIV-deficient cells accumulate succinate derived from glutamine" section:

"AOX acts as the sole surrogate terminal oxidase that accepts electrons directly from the coenzyme Q pool and thus partially restores ETS flux through complexes upstream of CoQ (Appendix Figure S1A). In a previous analysis of these cells, we observed the absence of assembled cytochrome c oxidase (COX) and a secondary reduction in complex I levels in the 6BKO cell line (Cunatova et al., 2024), resulting in impaired COX activity, abolished cellular respiration, and decreased NAD^+/NADH ratio. Overexpression of the alternative oxidase (AOX) partially rescued this phenotype (Cunatova et al, 2026; Cunatova et al., 2024). "

In addition, we included a new proof-of-concept experiment (Appendix Figure S3) to determine the PUFA stress response in 6BKO cells overexpressing the wild-type COX6B1 gene (6BRES, i.e. the addback model requested by the reviewer). While PUFA-enriched TGs and fatty acid desaturases levels were normalised, the level of GPX4 (similarly to 6BAOX) remained elevated. This indicates that 6BKO cells underwent metabolic adaptations that persisted even when AOX or COX6B1 was introduced into the KO background. We added the following paragraph to the Results "PUFA stress response protects cells from lipid peroxidation" part:

"As a proof-of-concept, we re-expressed the COX6B1 subunit in 6BKO cells (6BRES, Appendix Figure S3A) and assessed whether the PUFA stress response was normalised. TG levels were reduced to WT values (Appendix Figure S3B), and TG species with a high degree of unsaturation were comparable or even decreased (Appendix Figure S3C). Protein analysis (LFQ MS and WB analysis) revealed normalised expression of fatty acid desaturases; still, GPX4 remained elevated (as in 6BAOX cells), and iron metabolism enzymes were also altered (Appendix Figure S3D, E). Following 24 h of RSL3 treatment, lipid peroxidation tended to be even lower in 6BRES than in WT cells, although the difference did not reach statistical significance (Appendix Figure S3F). Cell proliferation under RSL3-induced peroxidative stress was comparable between 6BRES and WT at 24 h but was significantly improved in 6BRES at 48 h (Appendix Figure S3G), likely reflecting the persistently elevated GPX4, rather than a direct consequence of COX6B1 re-expression per se."

2. Are the effects of RSL3 on cell proliferation (Fig. 5) due to the already known lower proliferation capacity of COX6B1 KO cells? Using a cell death measurement instead of proliferation or metabolic activity would be more relevant to the conclusion. Also, including the relevant controls for lipid peroxidation evaluation with and without cotreatment with radical-trapping agents would be helpful.

We thank the reviewer for this point. Cell proliferation was chosen intentionally as a functional readout of the downstream consequences of altered lipid peroxidation. To control for the intrinsically lower proliferation capacity of 6BKO cells, all proliferation data were normalised to the vehicle-treated condition within each cell line. This key methodological detail was insufficiently highlighted in the original text. To clarify this, we added the following sentence to the Results "PUFA stress response protects the cells from lipid peroxidation" section:

“To directly assess the protective nature of the PUFA stress response, we induced lipid peroxidation with RSL3 and monitored cell proliferation relative to vehicle treatment over 24 hours (Figure EV2G-I) or 48 hours (Figure 3E-G).”

To show that cellular proliferation reflects cell death measurements, we performed a validation experiment and quantified dead cells as a percentage of propidium iodide-positive cells by flow cytometry in cells treated with vehicle, 1 µM RSL3 with or without 200 nM Ferrostatin-1:

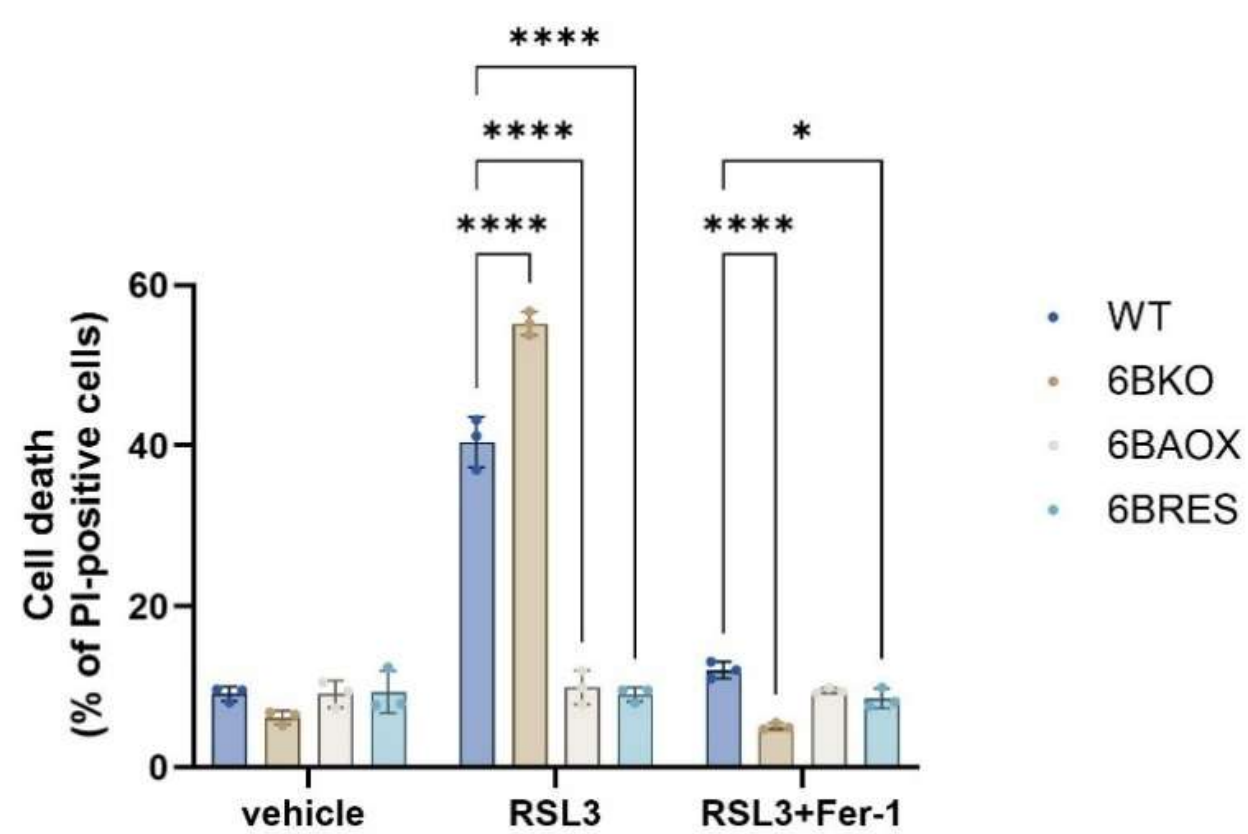

Cell death analysis in WT, 6BKO, 6BAOX and 6BRES cells. The cells were pretreated with 200 nM ferrostatin-1 (Fer-1) for 24 hours, followed by incubation with 1 µM RSL3 or vehicle (DMSO) for 24 hours. The cell death was measured by flow cytometry as propidium iodide (PI)-positive cells.

Compared with vehicle-treated controls, RSL3 significantly increased cell death in WT cells, with an even greater effect in 6BKO cells. Ferrostatin-1 fully rescued RSL3-induced cell death, confirming ferroptosis as the mechanism. As these results are fully consistent with the proliferation-based readouts already presented, and given the current figure count, we opted not to include this additional dataset in the manuscript.

3. Many of the conclusions are based on MS data without further validation. Therefore, it is relevant to evaluate mitochondrial activity in live cells by measuring oxygen and ATP levels. Additionally, quantifying mitochondrial number per cell, measuring cytochrome c oxidase activity, and determining the NAD+/NADH ratio in the cellular models are relevant to strengthen the manuscript.

We agree with the comment. The study focuses on metabolic adaptations of OXPHOS-limited cells, which were extensively characterised in our previous papers for standard parameters of mitochondrial function. However, this leads to an insufficient description here. To improve this, we included the following paragraph in the Results “CIV-deficient cells accumulate succinate derived from glutamine” chapter:

“In a previous analysis of these cells, we observed the absence of assembled cytochrome c oxidase (COX) and a secondary reduction in complex I levels in the 6BKO cell line (Cunatova et al., 2024), resulting in impaired COX activity, abolished cellular respiration, and decreased

NAD⁺/NADH ratio. Overexpression of the alternative oxidase (AOX) partially rescued this phenotype (Cunatova et al, 2026; Cunatova et al., 2024)."

Additionally, cellular respiration and glycolytic activity are now summarised in Figure EV3A, and changes in NAD^+/NADH are shown in Figure EV3G, to document mitochondrial activity in our models.

Minor points:

4. As observed in Fig. 1B, COX6B1 KO cells have increased doubling time compared to WT or AOX OE. Consider proliferation rate to perform flux analysis?

We thank the reviewer for raising this methodological point. The increased doubling time of 6BKO cells could indeed influence isotope incorporation if flux data were compared at a fixed time point without accounting for differences in proliferation. To address this, our isotope tracing experiments were designed to capture steady-state fractional enrichment rather than absolute flux rates. Cells were labelled for 24 hours (a duration sufficient to reach isotopic pseudo-steady state in both WT and 6BKO cells). Therefore, the observed differences in labelling patterns between WT and 6BKO cells reflect genuine metabolic reprogramming rather than a confounding effect of slower proliferation. To make this explicit, we have added the following sentence to the "Sample preparation for LC-MS analysis" section of Materials and Methods: "The cells were incubated in tracing medium for 24 hours to reach pseudo-steady-state labelling conditions, and data are expressed as a proportion of the total pool (fractional labelling) to account for differences in cell proliferation rates."

5. Accumulation of 2-hydroxyglutarate: measurement?

The steady-state levels of 2-hydroxyglutarate are now shown in Figure EV1B

6. TG accumulation: Are free fatty acids accumulated in both COX6B1 KO and AOX?

We thank the reviewer for this comment. We performed LC-MS analysis of the WT, 6BKO, 6BAOX cells and immortalised fibroblasts derived from healthy controls and patients with CIV deficiency (the data are now included in Figure 2B, S2A and 5B). Compared with WT, free fatty acid levels were elevated in 6BKO cells (and normalised in 6BAOX), but the difference was not significant due to high variation (Figure 2B). The following sentence was added to the Results "CIV-deficient cells accumulate triacylglycerols enriched for polyunsaturated fatty acids" section:

"*The levels of free fatty acids seemed to be increased; however, the difference was not significant (Figure 2B).*"

Moreover, the abundance of free PUFAs in 6BKO is elevated (Appendix Figure S2A). Significantly elevated levels of free fatty acids were also observed in both patient fibroblasts compared with control cells (Figure 5B). These results suggest that PUFAs are trafficked into TGs. The following sentence was added to the Results "CIV-deficient cells activate PUFA-mediated stress response" section:

"We further evaluated the abundance of free PUFAs, which may indicate their trafficking from membranes to TGs. In contrast to saturated or monounsaturated fatty acids, free PUFAs were elevated in KO relative to WT cells (Appendix Figure S2A). Upon AOX expression, free PUFAs and their sequestration to TGs were attenuated"

Further, in the Results "PUFA stress response is an adaptive mechanism in patients with mitochondrial deficiency" section, the next sentence was changed:

"Lipid class evaluation showed increased TG and free FA levels in the patient cells (Figure 5B), which also accumulated lipid droplets (Figure 5C)."

7. Lipid droplets measurement: explain the normalization of Fig. 3C, 6C, 7B, 8C

We modified the "Lipid droplet quantification" method by adding the following text:

"To normalise lipid droplet content for differences in cell number across images, nuclei were counted manually from maximum-intensity projections of the DAPI channel. The total BODIPY-stained area was then divided by the number of DAPI-counted nuclei in the same field of view, yielding a per-cell lipid droplet area (µm² per cell)."

The link to FIJI macro used for image processing was also provided in the method.

8. Relevant Mass spec data validation by immunoblotting.

To address this point, we performed SDS-PAGE/WB analysis of WT, 6BKO, 6bAOX and 6BRES using specific antibodies against SCD1 (Proteintech, 23393-1-AP) and GPX4 (Abcam, ab125066). Only the antibody against the GPX4 was specific, and the data showing a significant increase in 6BKO and 6BAOX compared to WT are now added in Figure S3F. Related to this, the new paragraph SDS-PAGE/WB analysis was added to Materials and Methods.

9. Statistics analysis in graphs of Fig. 7

We appreciate the comment. The data from Figure 7 are now in Figure 4E-G, and the statistical analysis has been corrected.

10. Improve read flow of the discussion section, focusing on the strength, novelty and implications of the presented data.

The discussion was rewritten, and the strength, novelty and implications of the findings are now emphasised.

Referee #3:

The manuscript examines how cells with impaired mitochondrial oxidative phosphorylation (OXPHOS) adapt lipid metabolism to preserve membrane integrity and avoid ferroptosis. Using genetic models of mitochondrial complex II, IV, and V deficiencies, hypoxia, patient-derived

fibroblasts, and plasma samples from individuals with MERRF syndrome, the authors identify a conserved "PUFA stress response" characterized by sequestration of polyunsaturated fatty acids (PUFAs) into triacylglycerols stored in lipid droplets, downregulation of fatty acid desaturases, and upregulation of GPX4. The study integrates metabolomics, lipidomics, isotope tracing, proteomics, and functional assays to propose PUFA sequestration as a protective adaptation in OXPHOS-compromised cells.

The authors have done a thorough job citing the existing literature.

Major Comments

The work provides a coherent and mechanistically plausible framework linking mitochondrial dysfunction to lipid remodeling as a defense against ferroptosis. Demonstrating that PUFA sequestration into triacylglycerols is conserved across multiple OXPHOS defects, hypoxia, and patient-derived fibroblast and blood, represents a meaningful conceptual advance with relevance to mitochondrial biology and regulated cell death.

A major strength is the complementary approaches and the use of multiple genetic OXPHOS-deficient models. Normalization of phenotypes by AOX expression in the CIV-deficient cells strengthens the causal link between impaired electron transport and lipid remodeling. The distinction between acute versus sustained OXPHOS dysfunction and its impact on fatty acid desaturation is well reasoned.

The functional assays using GPX4 inhibition, DGAT inhibition, ferrostatin-1 rescue, and fatty acid supplementation support the conclusion that PUFA sequestration protects against ferroptosis. While proliferation-based readouts are informative, inclusion of direct lipid peroxidation measurements would further strengthen the mechanistic link, particularly in the DGAT inhibition experiments.

We thank the reviewer for this valuable suggestion. To address this point, we measured lipid peroxidation directly using the fluorescent probe BODIPY 581/591 C11 (Thermo Scientific) in WT, 6BKO, and 6BAOX cells. Cells were treated with DGAT1 and 2 inhibitors, with RSL3 in the presence or absence of the ferroptosis inhibitor ferrostatin-1 (Fer-1), or with BSA-conjugated oleic acid (OA). The results are now presented in Figure 3D. Consistent with the proliferation-based readouts, RSL3-induced lipid peroxidation was elevated in WT cells and further increased in 6BKO cells. Both Fer-1 and OA supplementation effectively prevented RSL3-induced lipid peroxidation. Notably, pre-treatment with OA significantly attenuated RSL3-induced lipid peroxidation in 6BKO cells compared with WT, directly supporting the protective role of PUFA sequestration into TGs against ferroptosis. Related to this, the Results section: “PUFA stress response protects the cells from lipid peroxidation” was modified, and a new method (Lipid peroxidation analysis) was added to Materials and Methods.

The inclusion of patient-derived fibroblasts and plasma lipidomics from MERRF patients is an important strength. The elevated PUFA-enriched circulating triacylglycerols is intriguing, but

given cohort size and heterogeneity, these findings should be interpreted as hypothesis-generating and require validation in larger studies.

We agree with this comment and have modified the Discussion accordingly.

Minor Comments

Additional LC-MS methodological details and quality control metrics would improve reproducibility. Clarification of lipid droplet quantification normalization would be helpful. A brief definition of the term "PUFA stress response" early in the Results would help.

We thank the reviewer for these constructive suggestions. (i) To improve reproducibility, the “Metabolomic, lipidomic and fluxomic analysis” section in Materials and Methods has been significantly expanded to include additional details on instrument settings, conditions, and quality control metrics. (ii) Lipid droplet quantification normalisation is now described explicitly in the “Lipid droplet quantification” method section, and source code for the macro used in lipid droplet quantification is provided (see also response to Referee #1, Minor Point 7). (iii) The term “PUFA stress response” is now defined at its first appearance in the Results “CIV-deficient cells activate PUFA-mediated stress response” section:

”These results indicate that respiratory chain deficiency triggers a phenomenon we termed the PUFA stress response - an adaptive response that sequesters PUFAs from membranes into TGs stored in lipid droplets. It also includes the suppression of cellular desaturases and the upregulation of lipid peroxidation protective enzymes (GPX4).”

1st Revision - Editorial Decision 19th Jun 2026

Dear Dr. Pecinova

Thank you for the submission of your revised manuscript to EMBO reports. We have now received the full set of referee reports that is copied below.

As you will see, both referees are very positive about the study and recommend publication. Before I can accept the manuscript, I need you to address some minor points below:

- Your manuscript will be published in our Reports section. To make this possible, please combine the Results and Discussion section and keep an eye on our character limit: 27,000 characters (including spaces but excluding materials & methods and references).

- Please update the 'Conflict of interest' paragraph to our new 'Disclosure and competing interests statement'. For more information see
https://link.springer.com/journal/44319/submission-guidelines#conflictsofinterest

- Regarding the Author Contributions, we use CRediT to specify the contributions of each author in the journal submission system. Therefore, please remove the Author Contributions from the manuscript file and make sure that the author contributions in our online manuscript tracking system are correct and up-to-date. The information you specified in the system will be automatically retrieved and typeset into the article. You can enter additional information in the free text box provided, if you wish. See also our guide to authors https://www.embopress.org/page/journal/14693178/authorguide#authorshipguidelines.

- The information on funding provided in the manuscript and in the online system must be congruent. The European Union-Next Generation EU appears to be missing in the system.

- The correct titles for EV figures should be Figure EV1-Figure EV3, instead of Figure - Expanded view 1, etc.

- The callouts to S2A, S2B, C, S4, etc. are not correct. If these are Appendix figures, they need to be called out in full: Appendix Figure S#.

- Appendix: the author names and affiliations are not needed. Please provide a table of concent with page numbers and remove the blank page should be removed.

- The Appendix seems to have a rather low resolution and becomes pixelated when zooming in.

- Please remove the Reagents and Tools table from the manuscript and upload it as separate file. Our production team will typeset it into the article.

- BioRender should be acknowledged at the end of the Methods section in the following way:
Graphics:
(some of the... OR Figure #... OR synopsis) Graphics were created with BioRender.com.

- The manuscript sections should be in the following order: Title page - Abstract - Introduction - Results - Discussion - Methods - Data Availability - Acknowledgments - Disclosure Statement & Competing Interests - References - Figure Legends - (Main Tables with legends if applicable) - Expanded View Figure Legends.

- Materials and Methods should be Methods

- Please remove the image on page 18 (LIMEX workflow in the methods). If you feel that the workflow scheme is necessary, please let me know and we can discuss this further. You could e.g., include it in one of the main figures that show results derived from the workflow. If the approach has been published before, you can also refer to the previous publication.

- Please provide the specific URL for dataset (PXD069869) in the data availability statement.

- You state in the Data availability statement: "The source data of this paper are collected in the following database record: LINK". Please provide the missing link. If you refer to the source data uploaded to our online system, the statement is not necessary.

- Please address the following points in the figure legends and please highlight the changes:
- Please note that the figures 4C; EV-1C are mislabeled as figures 4D; EV-1B, respectively in the manuscript. This needs to be rectified.
- Please provide the exact p values in the legends of 1A-C,F,G,I; 2A-D; 3B,D,E,F,G; 4A,B,D,E,F; 5A-C,E-G; EV-1A-C,F; EV-2F-I;

EV-3B,D-I; Appendix Figure S1-B; Appendix Figure S3-C,E,G; Appendix Figure S4-A,B.
- Please specify whether "n" refers to technical or biological (independent) replicates in all figure legends.

- Figure 2C and others: please define the scale bar size only in the legend, not in the figure itself.

- The source data for immunofluorescent images would be more valuable if supplied as original files with metadata rather than a copy of the panel.

- Figure EV1E: please show the individual datapoints in addition to the mean and erro bars.

- In Figures EV3A (metabolic phenotype) and EV3G (NAD+/NADH ratio) you reused published data from cell lines 6BKO, 6BAOX, BETAKO and WT (Cunatova et el, 2024). While this reuse is fine as such, please carefully consider whether the WT control shown is appropriate since these data seem to have been obtained in different experiments done at different timepoints.

- Your response to the referees contains unpublished data in form of reviewer figures. We publish the referee reports and your response to these alongside the paper as Peer Review File. Please let us know in case you want to remove any of the reviewer figures. Please note that all data in the Peer Review File counts as published and that you would have to cite this document were your to re-use these in future publications.

- Please provide a short (1-2 sentences) summary of the findings and their significance in addition to the "Highlights" bullet points, and please upload these (summary and bullet points) as a separate Word file (type Cover Art/Synopsis image).

- Please provide the summary synopsis figure at a size of 550x300 pixels (width x height). Please note that the size is rather small and that text needs to be readable at the final size.

- As a standard procedure, we edit the title and abstract of manuscripts to make them more accessible to a general readership. Please find the suggested versions below my signature.

We look forward to seeing a final version of your manuscript as soon as possible.

With kind regards,

Martina

Martina Rembold, PhD
Senior Editor
EMBO reports

=================

Referee #1:

The authors have addressed our comments

Referee #3:

The authors responded to all my comments adequately.

=================

Polyunsaturated fatty acid sequestration protects against mitochondrial dysfunction-induced ferroptosis

Impaired energy production is a hallmark of mitochondrial oxidative phosphorylation (OXPHOS) defects. However, secondary metabolic disturbances also represent an important trigger for pathologies originating from OXPHOS aberrations. Cells with OXPHOS deficiencies accumulate triacylglycerols enriched in polyunsaturated fatty acids (PUFAs), which are stored in lipid droplets. Sequestration of PUFAs is a critical component of a broader stress response, which also includes downregulation of cellular desaturases and upregulation of glutathione peroxidase 4 (GPX4). Here, we demonstrate that this mechanism represents a physiologically relevant protective strategy, manifesting in cells under hypoxia and in immortalised fibroblasts derived from patients with primary mitochondrial complex IV deficiency. As proof of principle, we observe elevated PUFA-enriched triacylglycerols in the plasma of patients with Myoclonic Epilepsy with Ragged Red Fibres (MERRF). Our findings reveal a novel protective mechanism against ferroptosis, which preserves membrane integrity when mitochondrial respiration is compromised.

The authors have addressed all minor editorial requests.

2nd Revision - Editorial Decision 29th Jul 2026

Dr. Alena Pecinova
Institute of Physiology, Czech Academy of Sciences
Bioenergetics
Videnska 1083
Prague, Czech Republic 14220
Czech Republic

Dear Dr. Pecinova,

I am very pleased to accept your manuscript for publication in the next available issue of EMBO reports. Thank you for your contribution to our journal.

Your manuscript will be processed for publication by EMBO Press. It will be copy edited and you will receive page proofs prior to publication. Please note that you will be contacted by Springer Nature Author Services to complete licensing and payment information.

You may qualify for financial assistance for your publication charges - either via a Springer Nature fully open access agreement or an EMBO initiative. Check your eligibility: https://link.springer.com/journal/44319/how-to-publish-with-us

Should you be planning a Press Release on your article, please notify the production team via this form in order to coordinate publication and release dates: https://forms.cloud.microsoft/e/eH4A8JVP6g

If you have any questions, please do not hesitate to contact the Editorial Office. Thank you for your contribution to EMBO Reports.

Yours sincerely,

Martina Rembold, PhD
Senior Editor
EMBO reports

>>> Please note that it is EMBO Reports policy for the transcript of the editorial process (containing referee reports and your response letter) to be published as an online supplement to each paper. If you do NOT want this, you will need to inform the Editorial Office via email immediately. More information is available here: https://link.springer.com/partners/embo-press/editorial-policies#Peer%20review

EMBO Press Author Checklist

Corresponding Author Name: Alena Pecinova	
Journal Submitted to: EMBO Reports	
Manuscript Number: EMBOR-2025-63174-T	

USEFUL LINKS FOR COMPLETING THIS FORM
The EMBO Journal - Author Guidelines
EMBO Reports - Author Guidelines
Molecular Systems Biology - Author Guidelines
EMBO Molecular Medicine - Author Guidelines

Reporting Checklist for Life Science Articles (updated January
This checklist is adapted from Materials Design Analysis Reporting (MDAR) Checklist for Authors. MDAR establishes a minimum set of requirements in transparent reporting in the life sciences (see Statement of Task: 10.31222/osf.io/9sm4x). Please follow the journal's guidelines in preparing your
Please note that a copy of this checklist will be published alongside your article.

Abridged guidelines for figures
1. Data
The data shown in figures should satisfy the following conditions:
- the data were obtained and processed according to the field's best practice and are presented to reflect the results of the experiments in an accurate and unbiased manner.
- ideally, figure panels should include only measurements that are directly comparable to each other and obtained with the same assay.
- plots include clearly labeled error bars for independent experiments and sample sizes. Unless justified, error bars should not be shown for technical
- if n<5, the individual data points from each experiment should be plotted. Any statistical test employed should be justified.
- Source Data should be included to report the data underlying figures according to the guidelines set out in the authorship guidelines on Data

2. Captions
Each figure caption should contain the following information, for each panel where they are relevant:
- a specification of the experimental system investigated (eg cell line, species name).
- the assay(s) and method(s) used to carry out the reported observations and measurements.
- an explicit mention of the biological and chemical entity(ies) that are being measured.
- an explicit mention of the biological and chemical entity(ies) that are altered/varied/perturbed in a controlled manner.
- the exact sample size (n) for each experimental group/condition, given as a number, not a range;
- a description of the sample collection allowing the reader to understand whether the samples represent technical or biological replicates (including how many animals, litters, cultures, etc.).
- a statement of how many times the experiment shown was independently replicated in the laboratory.
- definitions of statistical methods and measures:
  - common tests, such as t-test (please specify whether paired vs. unpaired), simple χ2 tests, Wilcoxon and Mann-Whitney tests, can be unambiguously identified by name only, but more complex techniques should be described in the methods section;
  - are tests one-sided or two-sided?
  - are there adjustments for multiple comparisons?
  - exact statistical test results, e.g., P values = x but not P values < x;
  - definition of 'center values' as median or average;
  - definition of error bars as s.d. or s.e.m.

Please complete ALL of the questions below.
Select "Not Applicable" only when the requested information is not relevant for your study.

Materials

**Newly Created Materials**	**Information included in the manuscript?**	**In which section is the information available?** (Reagents and Tools Table, Materials and Methods, Figures, Data Availability Section)
New materials and reagents need to be available; do any restrictions apply?	Not Applicable	

**Antibodies**	**Information included in the manuscript?**	**In which section is the information available?** (Reagents and Tools Table, Materials and Methods, Figures, Data Availability Section)
For **antibodies** provide the following information:<br>- Commercial antibodies: RRID (if possible) or supplier name, catalogue number and or/clone number<br>- Non-commercial: RRID or citation	Yes	Reagents and Tool Table

**DNA and RNA sequences**	**Information included in the manuscript?**	**In which section is the information available?** (Reagents and Tools Table, Materials and Methods, Figures, Data Availability Section)
**Short novel DNA or RNA including primers, probes**: provide the sequences.	Not Applicable	

**Cell materials**	**Information included in the manuscript?**	**In which section is the information available?** (Reagents and Tools Table, Materials and Methods, Figures, Data Availability Section)
**Cell lines:** Provide species information, strain. Provide accession number in repository **OR** supplier name, catalog number, clone number, and/**OR** RRID.	Yes	Reagents and Tools Table, Materials and Methods
**Primary cultures:** Provide species, strain, sex of origin, genetic modification status.	Not Applicable	
Report if the cell lines were recently **authenticated** (e.g., by STR profiling) and tested for mycoplasma contamination.	Not Applicable	

**Experimental animals**	**Information included in the manuscript?**	**In which section is the information available?** (Reagents and Tools Table, Materials and Methods, Figures, Data Availability Section)
**Laboratory animals or Model organisms:** Provide species, strain, sex, age, genetic modification status. Provide accession number in repository **OR** supplier name, catalog number, clone number, **OR** RRID.	Not Applicable	
**Animal observed in or captured from the field:** Provide species, sex, and age where possible.	Not Applicable	
Please detail **housing and husbandry conditions**.	Not Applicable	

**Plants and microbes**	**Information included in the manuscript?**	**In which section is the information available?** (Reagents and Tools Table, Materials and Methods, Figures, Data Availability Section)
**Plants:** provide species and strain, ecotype and cultivar where relevant, unique accession number if available, and source (including location for collected wild specimens).	Not Applicable	
**Microbes:** provide species and strain, unique accession number if available, and source.	Not Applicable	

**Human research participants**	**Information included in the manuscript?**	**In which section is the information available?** (Reagents and Tools Table, Materials and Methods, Figures, Data Availability Section)
If collected and within the bounds of privacy constraints report on age, sex and gender or ethnicity for all study participants.	Yes	Materials and Methods

**Core facilities**	**Information included in the manuscript?**	**In which section is the information available?** (Reagents and Tools Table, Materials and Methods, Figures, Data Availability Section)
If your work benefited from core facilities, was their service mentioned in the acknowledgments section?	Yes	Acknowledgements

Design

Study protocol	Information included in the manuscript?	In which section is the information available? (Reagents and Tools Table, Materials and Methods, Figures, Data Availability Section)
If study protocol has been **pre-registered, provide DOI in the manuscript**. For clinical trials, provide the trial registration number **OR** cite DOI.	Not Applicable	
Report the **clinical trial registration number** (at ClinicalTrials.gov or equivalent), where applicable.	Not Applicable	

Laboratory protocol	Information included in the manuscript?	In which section is the information available? (Reagents and Tools Table, Materials and Methods, Figures, Data Availability Section)
Provide DOI OR other citation details if **external detailed step-by-step protocols** are available.	Not Applicable	

Experimental study design and statistics	Information included in the manuscript?	In which section is the information available? (Reagents and Tools Table, Materials and Methods, Figures, Data Availability Section)
Include a statement about **sample size** estimate even if no statistical methods were used.	Yes	Figures
Were any steps taken to minimize the effects of subjective bias when allocating animals/samples to treatment (e.g. **randomization procedure**)? If yes, have they been described?	Not Applicable	
Include a statement about **blinding** even if no blinding was done.	Yes	Materials and Methods
Describe **inclusion/exclusion criteria** if samples or animals were excluded from the analysis. Were the criteria pre-established? If sample or data points were omitted from analysis, report if this was due to attrition or intentional exclusion and provide justification.	Yes	Materials and Methods
For every figure, are **statistical tests** justified as appropriate? Do the data meet the assumptions of the tests (e.g., normal distribution)? Describe any methods used to assess it. Is there an estimate of variation within each group of data? Is the variance similar between the groups that are being statistically compared?	Yes	Materials and Methods

Sample definition and in-laboratory replication	Information included in the manuscript?	In which section is the information available? (Reagents and Tools Table, Materials and Methods, Figures, Data Availability Section)
In the figure legends: state number of times the experiment was **replicated** in laboratory.	Yes	Figures
In the figure legends: define whether data describe **technical or biological replicates**.	Yes	Figures

Ethics

Ethics	Information included in the manuscript?	In which section is the information available? (Reagents and Tools Table, Materials and Methods, Figures, Data Availability Section)
Studies involving **human participants**: State details of **authority granting ethics approval** (IRB or equivalent committee(s), provide reference number for approval.	Yes	Materials and Methods
Studies involving **human participants**: Include a statement confirming that **informed consent** was obtained from all subjects and that the experiments conformed to the principles set out in the WMA Declaration of Helsinki and the Department of Health and Human Services Belmont Report.	Yes	Materials and Methods
Studies involving **human participants:** For publication of **patient photos**, include a statement confirming that consent to publish was obtained.	Not Applicable	
Studies involving experimental **animals**: State details of **authority granting ethics approval** (IRB or equivalent committee(s), provide reference number for approval. Include a statement of compliance with ethical regulations.	Not Applicable	
Studies involving **specimen and field samples:** State if relevant **permits** obtained, provide details of authority approving study; if none were required, explain why.	Not Applicable	

Dual Use Research of Concern (DURC)	Information included in the manuscript?	In which section is the information available? (Reagents and Tools Table, Materials and Methods, Figures, Data Availability Section)
Could your study fall under dual use research restrictions? Please check biosecurity documents and list of **select agents and toxins** (CDC): https://www.selectagents.gov/sat/list.htm	Not Applicable	
If you used a select agent, is the security level of the lab appropriate and reported in the manuscript?	Not Applicable	
If a study is subject to dual use research of concern regulations, is the name of the **authority granting approval and reference number** for the regulatory approval provided in the manuscript?	Not Applicable	

Reporting

The MDAR framework recommends adoption of discipline-specific guidelines, established and endorsed through community initiatives. Journals have their own policy about requiring specific guidelines and recommendations to complement MDAR.

Adherence to community standards	Information included in the manuscript?	In which section is the information available? (Reagents and Tools Table, Materials and Methods, Figures, Data Availability Section)
State if relevant guidelines or checklists (e.g., **ICMJE, MIBBI, ARRIVE, PRISMA**) have been followed or provided.	Not Applicable	
For **tumor marker prognostic studies,** we recommend that you follow the **REMARK** reporting guidelines (see link list at top right). See author guidelines, under 'Reporting Guidelines'. Please confirm you have followed these guidelines.	Not Applicable	
For **phase II and III randomized controlled trials**, please refer to the **CONSORT** flow diagram (see link list at top right) and submit the CONSORT checklist (see link list at top right) with your submission. See author guidelines, under 'Reporting Guidelines'. Please confirm you have submitted this list.	Not Applicable	

Data Availability

Data availability	Information included in the manuscript?	In which section is the information available? (Reagents and Tools Table, Materials and Methods, Figures, Data Availability Section)
Have **primary datasets** been deposited according to the journal's guidelines (see 'Data Deposition' section) and the respective accession numbers provided in the Data Availability Section?	Yes	Data availability section
Were **human clinical and genomic datasets** deposited in a public access-controlled repository in accordance to ethical obligations to the patients and to the applicable consent agreement?	Not Applicable	
Are **computational models** that are central and integral to a study available without restrictions in a machine-readable form? Were the relevant accession numbers or links provided?	Yes	Materials and Methods
If publicly available data were reused, provide the respective **data citations in the reference list**.	Not Applicable	
